# Finding Potential Adverse Events in the Unstructured Text of Electronic Health Care Records: Development of the Shakespeare Method

**DOI:** 10.2196/27017

**Published:** 2021-08-11

**Authors:** Roselie A Bright, Summer K Rankin, Katherine Dowdy, Sergey V Blok, Susan J Bright, Lee Anne M Palmer

**Affiliations:** 1 US Food and Drug Administration Silver Spring, MD United States; 2 Booz Allen Hamilton McLean, VA United States; 3 US Food and Drug Administration Rockville, MD United States

**Keywords:** epidemiology, electronic health record, electronic health care record, big data, patient harm, patient safety, public health, product surveillance, postmarketing, natural language processing, proof-of-concept study, critical care

## Abstract

**Background:**

Big data tools provide opportunities to monitor adverse events (patient harm associated with medical care) (AEs) in the unstructured text of electronic health care records (EHRs). Writers may explicitly state an apparent association between treatment and adverse outcome (“attributed”) or state the simple treatment and outcome without an association (“unattributed”). Many methods for finding AEs in text rely on predefining possible AEs before searching for prespecified words and phrases or manual labeling (standardization) by investigators. We developed a method to identify possible AEs, even if unknown or unattributed, without any prespecifications or standardization of notes. Our method was inspired by word-frequency analysis methods used to uncover the true authorship of disputed works credited to William Shakespeare. We chose two use cases, “transfusion” and “time-based.” Transfusion was chosen because new transfusion AE types were becoming recognized during the study data period; therefore, we anticipated an opportunity to find unattributed potential AEs (PAEs) in the notes. With the time-based case, we wanted to simulate near real-time surveillance. We chose time periods in the hope of detecting PAEs due to contaminated heparin from mid-2007 to mid-2008 that were announced in early 2008. We hypothesized that the prevalence of contaminated heparin may have been widespread enough to manifest in EHRs through symptoms related to heparin AEs, independent of clinicians’ documentation of attributed AEs.

**Objective:**

We aimed to develop a new method to identify attributed and unattributed PAEs using the unstructured text of EHRs.

**Methods:**

We used EHRs for adult critical care admissions at a major teaching hospital (2001-2012). For each case, we formed a group of interest and a comparison group. We concatenated the text notes for each admission into one document sorted by date, and deleted replicate sentences and lists. We identified statistically significant words in the group of interest versus the comparison group. Documents in the group of interest were filtered to those words, followed by topic modeling on the filtered documents to produce topics. For each topic, the three documents with the maximum topic scores were manually reviewed to identify PAEs.

**Results:**

Topics centered around medical conditions that were unique to or more common in the group of interest, including PAEs. In each use case, most PAEs were unattributed in the notes. Among the transfusion PAEs was unattributed evidence of transfusion-associated cardiac overload and transfusion-related acute lung injury. Some of the PAEs from mid-2007 to mid-2008 were increased unattributed events consistent with AEs related to heparin contamination.

**Conclusions:**

The Shakespeare method could be a useful supplement to AE reporting and surveillance of structured EHR data. Future improvements should include automation of the manual review process.

## Introduction

### Background

Avoidable patient harm continues to be a significant problem [1]. To learn of adverse events (AEs), that is, patient harm, related to US Food and Drug Administration (FDA)–regulated products, the FDA relies on spontaneous reports from manufacturers, health care providers, and the general public. Published deficiencies of these reports [2-10] include nonstatistical representativeness of harm and problems. Now that electronic health care records (EHRs) are very common [11] and often more informative than billing codes from payment claims [7,12,13], we have an opportunity to leverage them for automated surveillance of patient harm [3,7,14,15]. We had two inspirations for naming the method after William Shakespeare: (1) in his play *Macbeth* [16], a king named Macbeth is surprised by an attack on his castle by soldiers camouflaged by trees, even though he had been warned that his downfall would come when the woods moved; and (2) scholars have been using word-frequency methods to discuss the true authorship of works from Shakespeare’s time [17].

### EHRs for Postmarketing Surveillance

Many methods for finding prespecified AEs in text [6,7,9,18-40] rely on predefining potential AEs (PAEs) before searching for prespecified words and phrases or manual labeling (standardization) by investigators. Crucially, events described in text may not necessarily be attributed to AEs [14,25,41]. We wanted to develop a method to identify PAEs, even if unknown or unattributed, without any prespecifications or standardization of notes.

There are many challenges to automated use of EHRs:

- Diagnosis codes may be “invalid, insensitive or non-specific” [20]
- “Often the notes contain medical and non-medical abbreviations, acronyms, numbers and misspelled words, which make it difficult to recognize the critical information in the notes. In other words, certain types of information such as ADEs [adverse drug events], indications, and signs and symptoms are harder to detect than other information such as drug names” [24]
- Medical entities in EHRs notes “can span across multiple words” [24]
- “… there is a lot of ambiguity among relevant named entities. Depending upon the context, the same exact phrase can be an ADE, indication, or a sign and symptom” [24]
- Periods do not always indicate the end of a sentence (“Dr.,” “1.23,” etc) [24]
- “…notes are frequently ungrammatical and are often inconsistently formatted. Ambiguity is common: MS, for example, can mean mitral stenosis or multiple sclerosis” [12]
- EHRs are “…subject to access restrictions…” [6]
- “…[N]ot all events and outcomes are consistently captured…” [15]
- We observed that different medical specialties, nurses, and other health care providers used different vocabulary.

We used the Medical Information Mart for Intensive Care III (MIMIC-III) EHR data set [42,43] because it is available to scientists with human subjects research training. MIMIC-III focuses on critical care in a major Boston teaching hospital. A published report using MIMIC-III noted [36]:

...several sentence segmentation tools available in popular NLP [natural language processing] toolkits, such as NLTK31 and spaCy, were tested and did not work well in clinical notes. In clinical notes, sentences do not always end with regular punctuation marks such as a period or question mark. More specifically, both regular punctuation marks and newline characters can serve as sentence breakers; however, newline characters can also be used for text wrap. Moreover, enumeration-like and list-like formats are also common in clinical notes, especially for physical exam and list of medications.

Many medical care AEs occur at higher frequency in hospital critical care settings and are related to complex illnesses, invasive procedures, and relatively long lists of treatments [44,45].

## General Methods

### Preprocessing

We used EHRs for critical care admissions within an adult hospital, the Beth Israel Deaconess Medical Center in Boston, MA. The Massachusetts Institute of Technology worked with the hospital to process EHRs from 2001 to 2012, including unstructured notes, into the MIMIC-III data set, which is publicly available to those meeting certification requirements. The research was designated as not human subjects research by the FDA Institutional Review Board under the Code of Federal Regulations, Title 45, Part 46 [46].

We removed admissions of patients aged <16 years and admissions without notes from the total of 58,976 hospital admissions, resulting in 49,284 admissions.

We noted during our initial manual review of the notes for dozens of admissions—to familiarize ourselves with the data—that discharge summaries did not include all PAE information in the progress notes. We decided to use all available notes for each study admission and created one document by concatenating them chronologically. The notes in the MIMIC-III database contained duplicated paragraphs, sentences, and lists. These duplications distort statistical analyses of terms used and hamper manual review of the notes. We applied the Bloatectomy package to remove the duplicate text from each admission document [47].

We removed the personally identifying information mask string and lowercased the text. We retained punctuation, numerals, and stop words because they convey clinical information and are sometimes components of abbreviations.

### The Shakespeare Method

The Shakespeare method has five steps:

1. Convert each document into a vector of n-gram (term) frequencies.
2. Create groups of vectors: target and comparison.
3. Extract terms in the target group that are significant for the target group.
4. Apply topic analysis to the target group–filtered vectors.
5. Review the original documents that have topic scores of interest to interpret the topics and find PAEs.

We have published the code [48].

**Figure 1:**
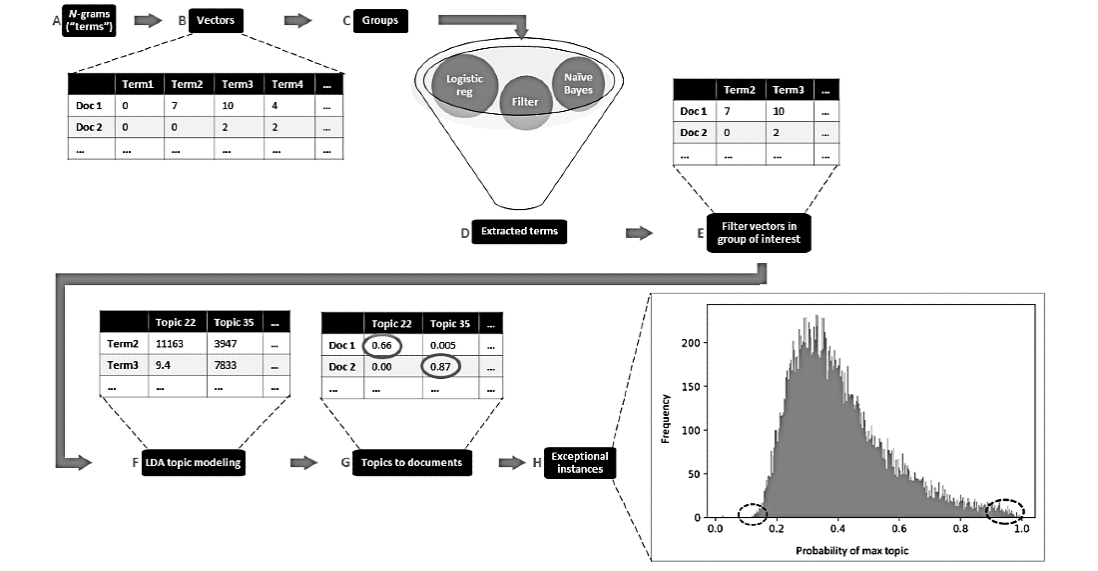
The Shakespeare method process with truncated examples. Step 1 (create n-gram vectors) includes (A) n-grams (terms) and (B) form vectors. Step 2 (create two groups) is (C) form groups. Step 3 (extract significant terms) is (D) extracted terms and (E) trim vectors in the group of interest. Step 4 (model topics) includes (F) latent Dirichlet allocation (LDA) topic modeling and (G) topics to documents. Step 5 (review topics) includes (H) identification of exceptional instances.

We selected two use cases to demonstrate the Shakespeare method: (1) comparing patients who received blood transfusion to those who did not and (2) comparing patient experiences in 1 year to the prior year. They shared step 1 (create n-gram vectors) of the Shakespeare method; we used the collocation detection skip-gram method for extracting the n-grams with n=1-5 consecutive words [49,50] (Figure 1A). We vectorized each document using a bag-of-words representation, where each dimension is represented by the frequency (count) of each n-gram (Figure 1B), resulting in a set of 7,422,044 words.

## The Transfusion Case

### Introduction

We decided to compare critical care patient admissions that involved blood transfusion (T) to comparison (C) admissions that had no transfusion events. An earlier version of the data set showed a higher risk of near-term mortality for patients receiving red blood cell transfusion compared to nontransfused patients [51]. By 2002, many transfusion AEs (TAEs) had been described [52]. During the time period covered by the data set, the transfusion research community recognized new TAE types—transfusion-related acute lung injury (TRALI) and transfusion-associated circulatory overload (TACO)—that prompted new guidelines to reduce the use of transfusion [53]. Simultaneously, far fewer reports were coming to the FDA than would have been expected, considering the level of professional concern [54-56].

### Study Objective

Our objective was to develop a method of using EHR notes to find recognized and unrecognized potential TAEs (PTAEs), which incidentally might also uncover other anomalies. We wanted our method to operate in the setting of the above-noted challenges.

### Methods

We followed step 1 (create n-gram vectors) as described in *The* *Shakespeare Method* subsection of the *General Methods* section.

#### Transfusion Case Step 2: Create Groups

We used the blood transfusion (n=21,443 admissions) and comparison (n=25,468 admissions) groups described in prior work [57] (Figure 1C).

#### Transfusion Case Step 3: Extract Significant Target Terms

**Figure 2:**
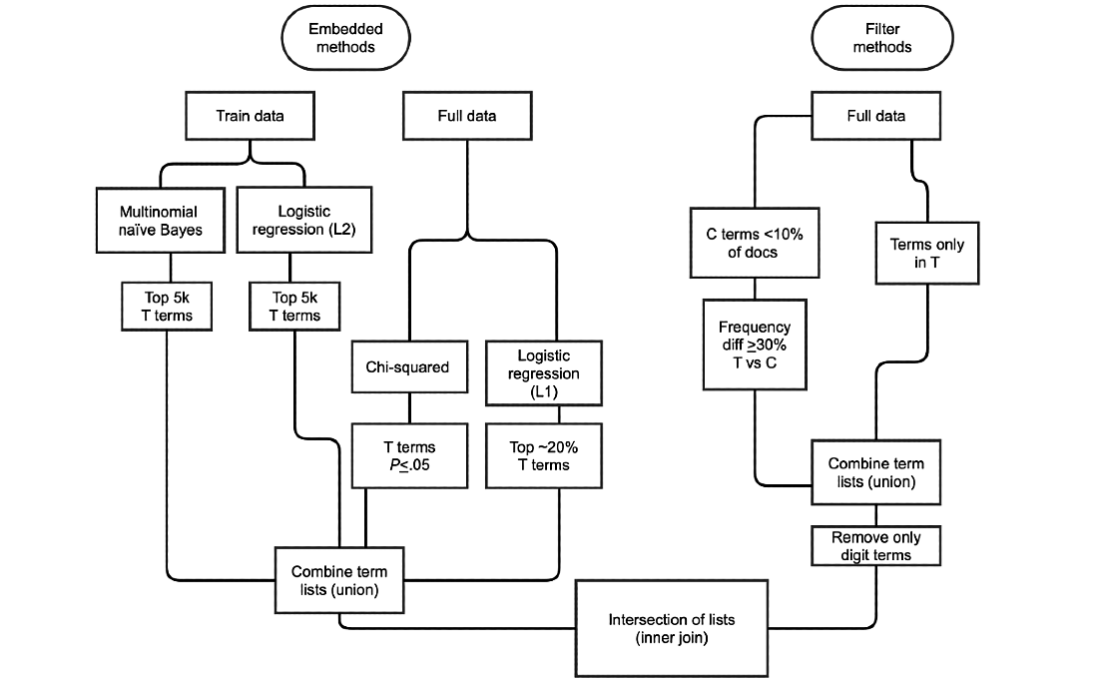
Flowchart of the embedded-based and filter-based term selection processes for the transfusion case. T: transfusion, C: comparison.

Our goal for steps 3 and 4 was to filter document vectors to only include terms that were significant to the transfused group and then model the topics within those terms in the transfused group to identify experiences emblematic of transfusion. We formalized the process of extracting these terms by looking at term coefficients associated with a classifier that learns to differentiate the two groups. We underwent an iterative process of trying multiple hyperparameters and classification models to identify these terms. We observed that an ensemble of two classification methods (naïve Bayes [NB] and logistic regression [LR]) and filtering [58-62] was useful for capturing common, infrequent, and rare terms that were significant for T. This term selection resulted in 41,664 terms (Figure 2). We reduced the T document vectors to include only the 41,664 terms (see Figure 1E for a truncated example).

#### Transfusion Case Step 4: Model Topics

**Figure 3:**
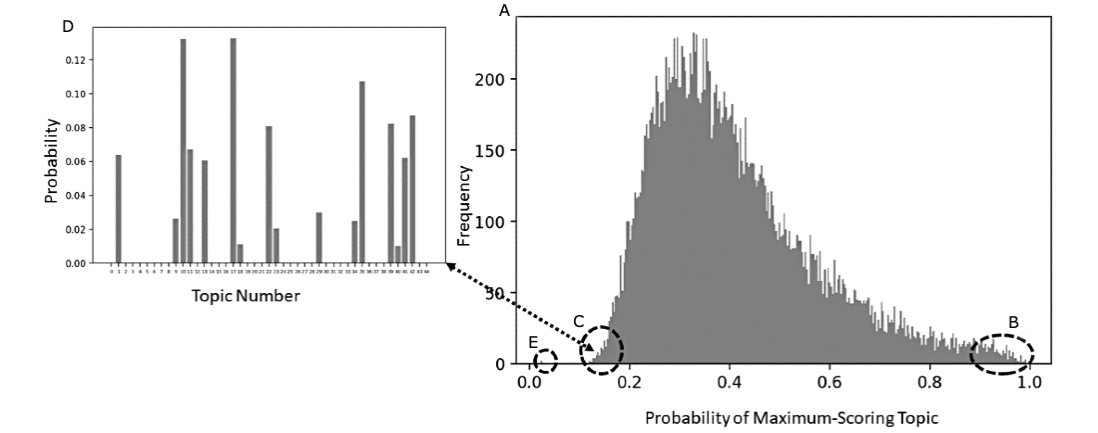
Topic-modeling results for the transfusion case (T): (A) distribution of all maximum document topic scores for all T, (B) documents that have only one strong topic, (C) documents that have many topics, (D) all topic scores for a single document that has multiple topics, and (E) two documents with a score of 0.022 for every topic.

Topic modeling is an unsupervised method commonly used in NLP to extract the most relevant terms for each topic (cluster) of similar documents [63,64]. We chose latent Dirichlet allocation (LDA) [65] to accomplish topic modeling of the T documents. LDA is a generative probabilistic model that results in interpretable dimensionality reduction, which means that we reduced 41,664 terms to 45 topics for our data. A topic is a multimodal distribution of terms over an entire vocabulary (in our case, all the filtered terms). A topic consists of co-occurring terms in this corpus of T documents. Each document can have a mixture of these topics. Each topic contribution in a document is a probability (we refer to this as a document topic score); thus, the scores of all topics for a document sum to 1 (Figure 3D).

We performed topic modeling (Figure 1F,G) by applying the LDA model to the filtered document word vectors (Figure 1E) to find co-occurring terms and group them into topics.

Topic modeling resulted in a matrix of scores for each term by each topic, which we refer to as term scores (Figure 1F). An additional matrix shows the probability of fit for each topic (Figure 1G).

Figure 1G shows the topic document scores, and the maximum topic for each document is circled. This maximum topic is the topic that is the strongest for a document. When the maximum topic score is low, we can infer that the document fits many topics, which in critical care could mean that the patient has many clinical issues, some of which might be PTAEs and should be reviewed.

The maximum document topic scores distribution was plotted in the maximum topic histogram shown in Figure 3A. There were few documents in this corpus with a high maximum topic probability score (Figure 3B, right tail). Most of the documents were comprised of two or more topics (6.1 was the mean number of topics with a minimum score of ≥0.03).

A small number of documents in the left tail of Figure 3C had a low (<20%) maximum topic probability score, meaning that these documents were comprised of many topics. This was further illustrated in the inset (Figure 3D) displaying the topic distribution of a single document from this left tail, which had multiple topics. These extreme documents in the right and left tails were selected for manual review.

An important consideration for LDA is that the number of topics must be selected a priori. The results of topic modeling change depend on the number of topics assigned to a corpus—this is an iterative (hyperparameter tuning) process that requires human judgment to interpret the topics (based on the top terms in each topic) and determine which number of topics best fits the corpus. With too few topics assigned, topics are not cohesive and do not add any clarity or information to an analysis. With too many topics assigned, “incoherent” topics that do not capture terms common to the member documents proliferate; additionally, useful topics are likely split among smaller, more specific topics, although that does not limit the ability to analyze true clusters in the corpus.

To tune the hyperparameters of the LDA model, we calculated models with the following numbers of topics: 25, 35, 45, 55, 65, 75, and 85. We observed (data not shown):

- As the number of topics rose, at first, clinically meaningful topics were added. Still, at higher numbers, the additional topics were incoherent, and the large, meaningful topics tended to split in ways that were not meaningful.
- The top words in topics were generally consistent for topics that were alike across multiple topics. For example, a mechanical ventilation topic was present whether the topic number was 9, 10, or 26.
- Although particular documents changed, the documents with high top topic scores had the top topic terms.
- Topics that had high document topic scores had overlapping concepts in the highest-scoring terms.
- Several topics were difficult to interpret and had low maximum values for both word scores and document topic scores.
- There were 1 to 2 dozen known TAEs [66,67].
- Many documents had several topics, reflecting the clinical complexity of patients in the critical care unit [68].

#### Transfusion Case Step 5: Review Topics

To evaluate whether topics described PTAEs, we selected the following records for manual document review: the three top-scoring documents for each of the 45 topics (Figures 1H and 3A,B), the 7 documents with the most topics with significant scores (≥0.03) (such as in Figure 3C), and 24 randomly selected documents from the T group. We abstracted events, observations, clinicians’ attributions of causality, and clinicians’ diagnoses, as well as their dates (where offered). We used further abstractions and tabulations to protect patients’ confidentiality.

We tested comparisons with the Fisher exact test [69].

### Results

Despite the inclusion of n-grams with a length of 1 to 5 in the vectorization, the terms that we extracted during classification were unigrams.

#### Distribution of Transfusion Topic Document Scores

A histogram of maximum topic scores (Figure 3A) showed the distribution of each document’s maximum (strongest) topic. There were few documents in this corpus with a high maximum topic probability score (Figure 3B, right tail). The left tail of Figure 3C shows a small number of documents with a maximum topic probability score that is low, or less than 20%, suggesting these documents comprised many topics. Figure 3D illustrates this with the topic distribution of a single document from this left tail. The lowest maximum topic document score was 0.022. Two documents had topic document scores of 0.022 for every topic (Figure 3E). They each had only one short record: a brief electrocardiogram report.

**Figure 4:**
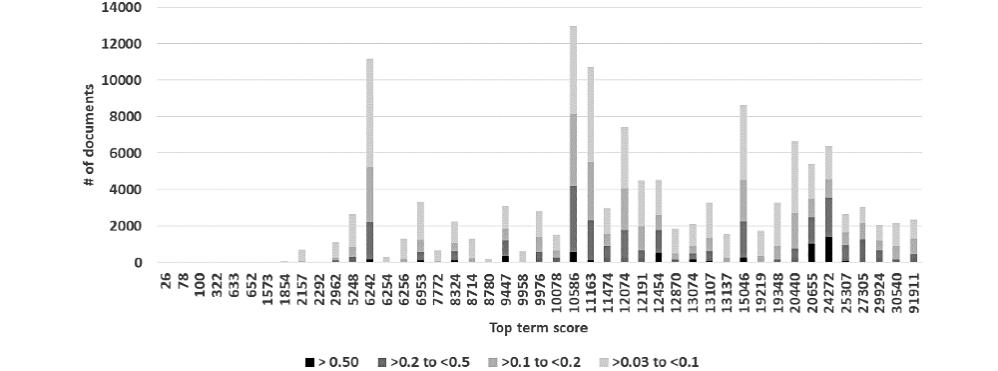
Distribution of topic document scores and top term scores for the transfusion case.

There was no strict relationship between top word score and the frequency distribution of document topic scores (Figure 4). Table 1 shows the categories of maximum document topic scores per number of topics. It shows that if there is one topic, the score is over 0.50. As the number of topics increases, the maximum topic score declines. The average number of topics with a topic document score >0.03 was 6.1. The maximum topic document score was 0.994.

**Table 1:** Maximum document topic score in the transfusion case for documents in relation to number of topics in a document.

Number of document topic scores ≥0.03	Maximum document topic score, n		
	Score≥0.5	0.2≤score<0.5	0.1≤score<0.2
0	0	0	0
1	132	0	0
2	484	13	0
3	1121	326	0
4	1462	1138	0
5	1179	2582	0
6	610	3509	13
7	209	3595	85
8	55	2427	191
9	13	1183	265
10	0	414	173
11	0	113	91
12	0	25	25
13	0	2	5
14	0	0	1

#### Top-Scoring Documents for Each Transfusion Topic

Table S1 (Multimedia Appendix 1) shows, for each topic, the score for the top term, the top 20 terms, the top document score, and the distribution of documents by document score range. The rows are sorted by top document score. The maximum word score ranged from 26 to 91,911. The terms with the top 20 scores included plain English words, clinical words, acronyms, shortened words, and misspellings. The maximum document score for a topic went as high as 0.994. The document scores were widely distributed.

Table S2 (Multimedia Appendix 1) presents the summaries of 135 documents. As is expected when hyperparameters of the model are optimal, most topics (n=35) were “coherent,” meaning the top documents had clear common themes within topics consistent with the lists of the top 20 terms in the topic. The coherent topics had higher top document scores and tended to be the maximum-scoring topics. Among the least coherent topics, the tendency for documents was to have some other topic as the maximum-scoring topic. This is expected with LDA, as the words that do not fit into a coherent topic will be allocated to separate “junk” topics.

The tabulation of the presence or absence of the notes expected to have the most clinical information showed that 122 had a discharge summary, 66 had a nursing note, and 21 had a physician progress note. None of the documents attributed an AE to transfusion in the billing codes.

New or worsening PTAEs occurring within 1 to 2 days in the T group were:

- In the heart category: atrial fibrillation, tachycardia, bradycardia, other heart rhythm abnormalities, hypotension;
- In the lung category: hypoxia, mechanical ventilation, bilateral pleural effusions, pulmonary edema;
- In the volume category: edema, diuresis therapy, acute kidney failure;
- In the absence of evidence for other infections: fever or chills.

Many documents (n=40) could not be evaluated for TAEs because either the transfusion dates were missing or there was no identified treatment when transfusion could be presumed. For others, there was a clear alternate reason for heart or lung problems: advanced cancer (n=7), thrombotic thrombocytopenic purpura present at admission (n=1), liver failure (n=1), and lung infection (n=1).

Out of the remaining 85 documents with transfusion data, 52 had evidence of PTAEs; the most common were heart PTAEs (n=35) and lung PTAEs (n=33), while non–infection-related fever or chills (n=12) and fluid overload (n=12) were less common. A few documents explicitly considered transfusion as the cause of AEs: in topic 30 (blood disease), one attributed disseminated intravascular coagulation to transfusion and another listed but discarded the possibility of TRALI or TACO, a document in topic 3 (bone trauma from motor vehicle accident) proposed PTAEs, and a document in topic 40 attributed a drop in platelets to transfusion. In 2 documents, the PTAEs were attributed to contrast (topic 37, kidney failure), a brand name for metronidazole (topic 38, colon problem), and surgery (3 cases of bone trauma from a motor vehicle accident).

Documents with transfusion timing but no apparent TAE were in the following topics: 10 (one of the mechanical ventilation topics), 2 (esophageal varices banding), 7 (spine surgery), 18 (gastrointestinal bleeding), 31, and 8. For 10 documents, separate transfusion and PTAE codes were present but were not conceptually linked.

We read 24 randomly selected documents to obtain 20 that did not have advanced cancer, cirrhosis, or severe lung trauma. They are summarized at the bottom of Table S2 (Multimedia Appendix 1).

The documents in the cardiovascular topic group were more likely than the random group to have any of the heart PTAEs (proportion difference=0.47; *P*=.02). The analogous analysis for 14 documents in the lung failure topic group showed a higher rate of any lung PTAEs (proportion difference=0.37; *P*=.049).

Table S3 (Multimedia Appendix 1) depicts the characteristics of the 8 documents that had 13 or 14 topics. Their document topic scores were distributed across many topics, and the notes described a large number of medical challenges to the patients. All of these documents had both discharge summaries and nurse progress notes. One physician wrote that the patient developed alloantibodies and had a delayed transfusion reaction. None of the billing codes linked transfusion to an AE, and in 2 records, the codes included an outcome code. All 8 documents provided dates of transfusion, including 3 for which cancer was the more likely cause of the AE. Of the remaining 5 documents, 3 had pulmonary PTAEs:

- The document with all three types of PTAEs had only one topic with a score above 0.1 (topic 42, heart attack), and the notes, but not codes, indicated the patient had a delayed transfusion reaction.
- The document with pulmonary and volume PTAEs had the following topics with scores ≥0.1: topic 42 (heart attack), topic 24 (tPA [tissue plasminogen activator] to lyse thrombus), topic 10 (cirrhosis), and topic 1 (x-ray confirmation of device placement). The notes attributed worsening acute kidney failure to an antibiotic.
- The document with only pulmonary PTAEs had the following topics with scores ≥0.1: topic 24 (tPA to lyse thrombus), topic 10 (mechanical ventilation), and topic 37 (kidney failure).

### Discussion

The Shakespeare method successfully identified PTAEs. The three top-scoring documents in cardiovascular topics (topic 17, heart valve repair; topic 33, tapped pericardial effusion; topic 35, coronary artery bypass graft; topic 42, heart attack; and topic 11, vascular repair) were associated with cardiovascular PTAEs: atrial fibrillation, tachycardia, bradycardia, other heart rhythm abnormality, or hypotension, which are features of TAEs [66,67].

Mechanical ventilation and nitric oxide therapy (topics 9, 10, 16, and 26) were used to treat lung failure [70], which was also a topic (topic 29, acute respiratory distress syndrome). The associated breathing PTAE (hypoxia, mechanical ventilation, bilateral pleural effusion, and pulmonary edema) are components of TRALI and TACO [66,67].

Other PTAEs that correspond with known TAEs were also observed in the top three documents of topics:

- Features of the volume overload component of TACO (edema, acute renal failure, and diuresis) [67];
- A feature of hemolytic transfusion reaction and febrile nonhemolytic transfusion reaction (fever without other signs of infection) [67].

#### Distribution of Transfusion Topic Document Scores

**Figure 5:**
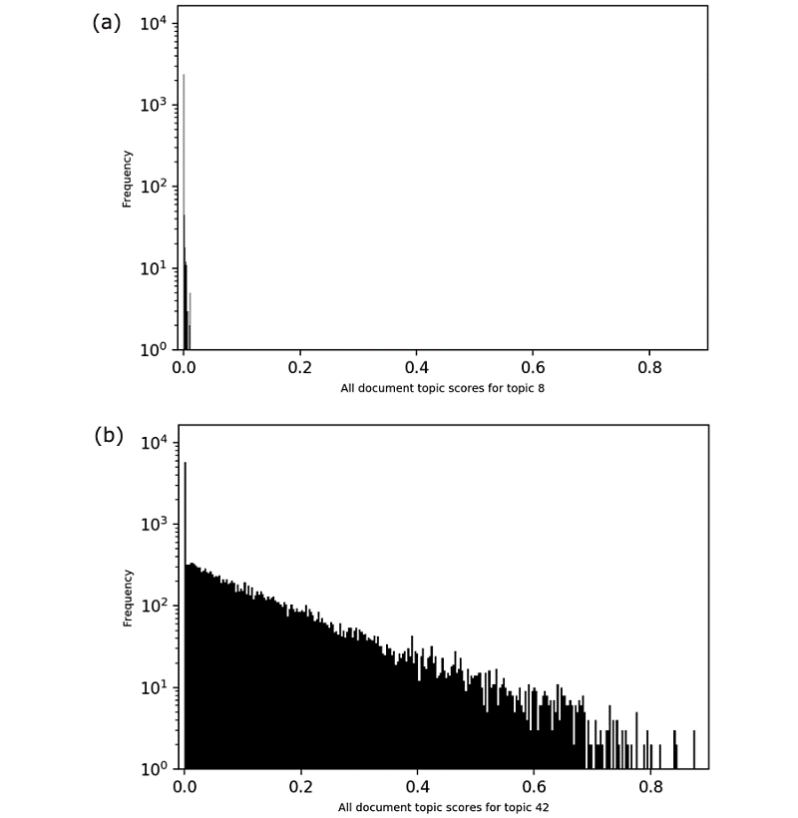
Distribution of document topic scores for two topics in the transfusion case: (a) topic 8, a noncoherent topic, and (b) topic 42, a coherent topic.

Incoherent topics had few or no documents with high topic document scores; most documents scored at or close to zero (see example in Figure 5A). A coherent topic follows a similar distribution, but the range is much greater, as seen in the x-axis of Figure 5B when compared to Figure 5A. The coherent topics received higher scores in many documents.

#### Top-Scoring Documents for Each Transfusion Topic

Many topics were conditions that can be reasons for transfusion: anemia [68]; heart attack [71]; blood disease (including blood cancers, chemotherapy, bone marrow transplant, neutropenia, thrombocytopenia, and pancytopenia) [68,72]; major surgery, vascular occlusion or repair, and gastrointestinal problems or bleeding [73]; and tPA to lyse thrombus, because antithrombotic treatment can cause bleeding [74].

Some topics could be consequences of the reasons for transfusion. Tapped pericardial effusion is a candidate because pericardial effusions can result from cancers, heart disease, aortic dissection, and other conditions [75] that prompt transfusion [76]. Past sternotomy, a consequence of heart surgery [77], is often a reason for transfusion [78]. Pneumomediastinum could be caused by surgery, or tearing of the esophagus or trachea [79], which in turn could be a reason to transfuse [73]. Skin breakdown can be a consequence of long-term bed rest [76,80], which is generally associated with critical illness and anemia [68], which in turn prompts transfusion [68].

Some could be alternate reasons for a PTAE: advanced cancer [81], liver disease [82], and infection [83].

Others could be a PTAE or sequelae of PTAEs: mechanical ventilation, which is a known consequence of TAEs [84,85]; pneumomediastinum, which could be caused by mechanical ventilation [79]; a tracheostomy tube, which is placed when long-term mechanical ventilation is anticipated [86]; acute respiratory distress syndrome, which shares features (noncardiogenic pulmonary edema and hypoxia) with TRALI [84] and is also known as acute lung injury and is treated with noninvasive or invasive ventilation [87]; and permanent hemodialysis indicating permanent kidney injury [88], which can result from hemolytic transfusion reactions [89] and is associated with volume overload [90], which is part of TACO [66].

#### Documents With Multiple Transfusion Topics

The high number of topics per document reflects the complexity of patients in the critical care unit. Multiple topics covering illnesses and procedures were expected for critically ill patients and were the norm for the vast majority of documents. The documents with 13 and 14 significant topics described many complex clinical problems consistent with the need for critical care. Several of the documents had a variety of PTAEs in more than one category, suggesting the importance of checking the documents with multiple nontrivial topics for PTAEs.

## The Time-Based Case

### Introduction and Study Objective

We wanted to simulate real-time analysis to find new or increasing events in the most recent time period. We examined whether the Shakespeare method would overcome the challenges of EHR texts to detect not only clinical and administrative changes but also trending PAEs, including those related to heparin contamination, which were first reported early in 2008 [91]. Heparin is an anticoagulant used in surgeries [91].

### Methods

The MIMIC-III EHRs for critical care admissions used one medical record system from 2001 to 2008 and another system post-2008. We received the real dates, within several weeks, for the earlier data. We followed the same step 1 (create n-gram vectors) as described in *The Shakespeare Method* subsection of the *General Methods* section.

#### Time-Based Case Step 2: Create Groups

We then divided the study population into three cohorts: admissions starting between July 1, 2001, and June 30, 2006 (period 1; 14,410 documents); July 1, 2006, to June 30, 2007 (period 2; 3581 documents), and July 1, 2007, to June 30, 2008 (period 3; 3296 documents).

#### Time-Based Case Step 3: Extract Significant Target Terms

**Figure 6:**
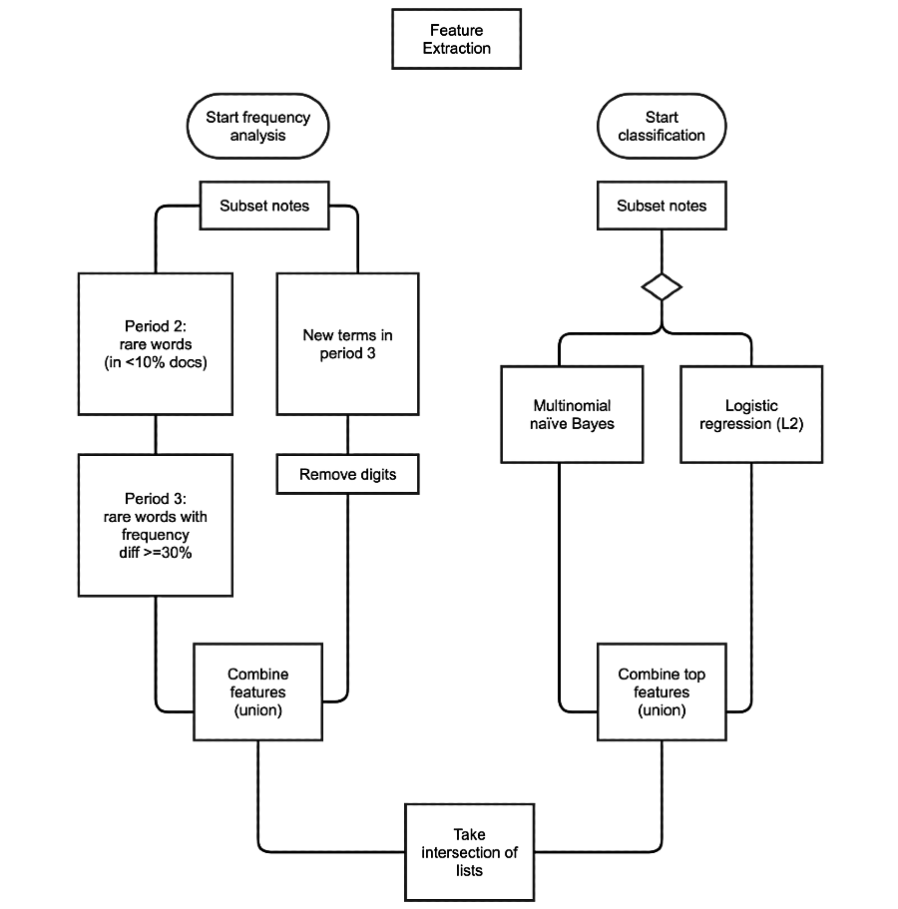
Feature extraction flowchart for the time-based case. This demonstrates the two parallel processes for extracting relevant features prior to topic modeling on the notes: term frequency analysis and binary classification of notes.

To focus on new or increasing AEs, we reduced the number of words to analyze by filtering by whether they were unusual and increasing (or new) in period 3 compared to period 2 (Figures 1C,D and 6A). We adopted two parallel approaches, as shown in Figure 6: (1) binary classification of the notes and (2) analysis of term frequency between periods 3 and 2.

For the binary classification, we fit two classification models: LR with L2/ridge regularization [61] and multinomial NB [59,60]. Model evaluation found LR outperformed NB (with a weighted average F1 score of 0.76 compared to NB’s weighted average F1 of 0.69), but that NB more effectively identified completely new terms in the target time period.

After evaluating the models, we refit both models without a train-test split on the entire 24-month data set and combined the top 5000 features from LR (those with the highest positive coefficient associated with the positive target class) and the top 5000 features from NB (those with the lowest log probability ratio). Combining the lists resulted in a set of 9896 terms.

We used frequency analysis to find emerging rare clinical events. We identified two groups of terms: (1) those which appeared in fewer than 10% of documents in period 2 and saw a 30% increase in raw frequency in period 3, and (2) any terms that never appeared in period 2 and did appear in period 3. For those new terms appearing in period 3, we filtered out digit-only terms (a large number of terms in this group).

For the final feature set, we took the intersection of terms identified from the binary classification and frequency analysis processes. This resulted in 6122 significant terms identified from the initial 117,049 unique terms in the documents from period 3 (5.2% of terms). We revectorized (Figure 1E) the 12-month corpus from period 3 using the combined feature list as our vocabulary (which has the effect of filtering the notes to only include terms in the vocabulary).

#### Time-Based Case Step 4: Model Topics

The co-occurrence of words in documents in the last time period was analyzed with LDA topic analysis [65]. We chose the final number of topics (n=20) based on a balance of large and small topics and at least one topic with no substantive words. We used the words with the highest scores of their relationship to topics (Figure 1F), as well as the topic document scores that indicate the probability of the topic fit for a document (Figure 1G), to explore topic meanings. We manually read the three top-scoring documents for each topic (Figure 1H).

#### Time-Based Case Step 5: Review Topics

Documents from selected individual admissions, as well as summary data from July 2001 to June 2008, were used to evaluate whether any topics formed around AEs. Most topics inspired time plots of selected words, diagnosis codes, or procedure codes (see criteria in Table S4, Multimedia Appendix 1) through periods 1, 2, and 3. Slopes were analyzed for changes [92,93].

For this report, out of concern for patient privacy, we substituted generic words (such as “condition01,” “condition02,” etc) for rare conditions, drugs, events, and languages since the year of admission is being presented. Related substitute words (eg, “condition09a,” “condition09b”) were used as synonyms.

### Results

Table 2 shows the statistics for each topic. The strength of the maximum word score in a topic roughly corresponded with the number of admissions that had strong matches with the topic. The words in many of the topics seem to readily suggest interpretations, for example, long complex stay (topic 18), heart problem (topic 3), trauma (topic 19), cardiac catheterization (topic 7), brain (topic 1), cardiac catheterization (topic 17), abdomen (topic 12), uterus (topic 16), and a foreign language (topic 2). The other topics were deemed broad.

**Table 2:** The score for the top term, top 20 substantive terms, top document score, and distribution of documents by document score range for each topic in the time-based case. “Substantive” terms had topic scores above the minimum topic score.

Topic #	Top term score	Top 20 substantive terms	Top document score	Documents in topic score range, n				
				≥0.03	≥0.5	≥0.2 to <0.5	≥0.1 to <0.2	≥0.03 to <0.1
18	75,372	for, hr, plan, vent, intubated, cont, today, skin, are, family, per, support, increased, off, goal, iv, placed, trach, foley, pain	0.99	1793	505	623	326	339
3	42,070	for, hr, pain, bp, are, you, iv, family, time, ccu, per, sats, note, heart, micu, received, skin, if, acute, plan	1.0	2224	912	697	328	287
19	39,731	for, are, pain, you, comparison, acute, upper, evaluate, iv, trauma, hospital, if, note, time, large, level, pleural, wbc, read, throughout	1.0	2089	355	880	468	386
7	30,722	for, are, pain, pleural, cabg, hr, plan, per, comparison, off, bp, pericardial, time, neo, iv, heart, md, mm, mr, catheter	1.0	1686	589	321	319	457
1	12,352	for, are, family, subarachnoid, mm, comparison, pain, iv, occipital, sdh, large, evaluate, plan, cont, acute, craniotomy, per, hr, note, goal	1.0	749	181	235	118	215
4	3523	catheter, pleural, for, pain, jp, [pain-reliever], placed, large, into, pigtail, hr, cont, french, increased, are, pseudoaneurysm, upper, skin, iv, comparison	0.54	683	1	75	180	427
17	3462	for, are, mca, into, time, catheter, arteriogram, occlusion, mm, acute, french, ica, iv, placed, territory, large, cont, comparison, goal, family	0.77	534	39	99	127	269
12	216	[condition01], section, gynecology, [condition02], dystrophy, cesarean, [anti-thyroid], transabdominal, [event01], lmp, wk, [procedure01], [progesterone], prenatal, [condition03], [condition04], [antispasmodic], enteropathy, [condition05], [condition06]	0.22	31	0	1	7	23
11	75	pentobarb, pentobarbital, cmv, encasement, prison, [condition07], satellite, hematologic, rent, [condition08], [condition09a], [condition09b], [antibiotic], federal, bleach, [device01], allergic, [rare-word01], cluster, [rare-word02]	0.11	26	0	0	1	25
5	63	[rare words, misspelled words]	0.05	1	0	0	0	1
15	36	[rare words, misspelled words]	0.13	2	0	0	2	0
16	15	[rare words, misspelled words]	0.11	2	0	0	1	1
6	14	[rare words, misspelled words]	0.02	0	0	0	0	0
10	11	[rare words, misspelled words]	0.06	2	0	0	0	2
0	10	[rare word]	0.04	1	0	0	0	1
2	9	[rare words, foreign language words, misspelled words]	0.12	3	0	0	1	2
14	8	[rare words, misspelled words]	0.03	1	0	0	0	1
9	7	[rare words, misspelled words]	0.07	2	0	0	0	2
13	6	[rare words, misspelled words]	0.06	3	0	0	0	3
8	0	—^a^	0	0	0	0	0	0

^a^Not applicable.

#### Common Topics for the Time-Based Case

For the most common topics, the admissions with the top three topic match scores are summarized in Table S5 (Multimedia Appendix 1). For the topics with words that suggested an interpretation, the records supported the interpretations. For the other topics, the records suggested interpretations that were consistent with the top words. Each of the three top-scoring admissions within a topic were quite similar to each other (an indication that the topics were coherent and the model was working correctly, with the exception of the third admission in topic 3).

The three top-scoring documents for topic 18 described long complex stays, which included large numbers of notes. The general words in the topic (“for,” “hr,” “plan,” “cont,” “today,” “skin,” and “are”) were nearly ubiquitous in periods 2 and 3. The words indicating mechanical ventilation (“vent,” “intubated,” and “trach”) were present in between 51% and 58% of the admissions per quarter in periods 2 and 3, with a slight, clinically insignificant increase for period 3. The lengths of stay and numbers of notes also did not vary between periods 2 and 3.

We noticed that among the five records in Table S5 (Multimedia Appendix 1) that mentioned cardiac catheterization, all mentioned explicit or implied dosing with heparin followed the same day with hypotension that required treatment (heparin is generally part of cardiovascular procedures) [94].

**Figure 7:**
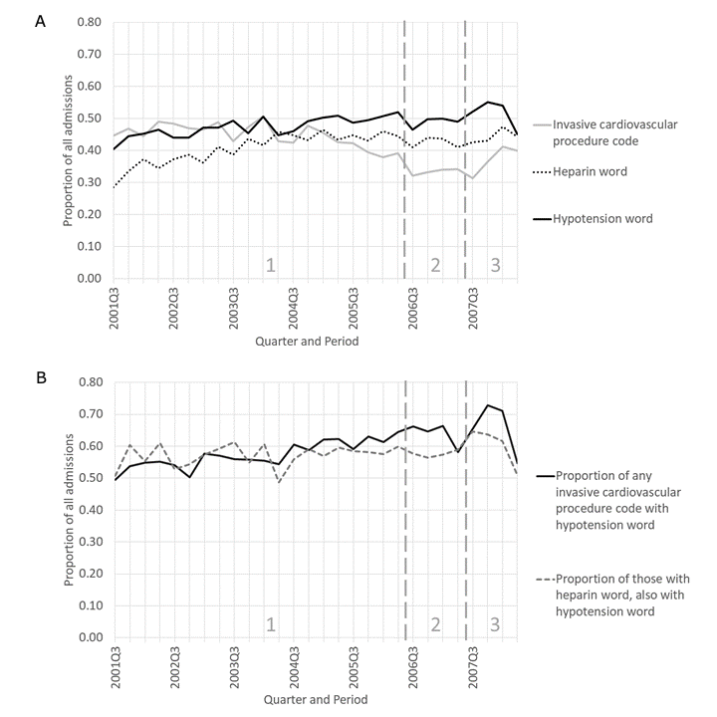
Heparin and hypotension for the time-based case (see Table S4 [Multimedia Appendix 1] for search criteria details). (A) Invasive cardiology-, heparin-, and hypotension-related criteria as a proportion of all admissions. Invasive cardiology is presumed to involve heparin treatment. For invasive cardiovascular procedure code, slope=–0.0053 (95% CI –0.0069 to –0.0037), *P*<.001; for heparin word, slope=0.0039 (95% CI 0.0025-0.0054), *P*<.001; and for hypotension word, slope=0.0029 (95% CI 0.0017-0.0040), *P*<.001. (B) The word “hypotension” as a proportion of presumed heparin exposure. For the proportion of any invasive cardiovascular procedure code (presumed to involve heparin), slope=0.0055 (95% CI 0.0038-0.0072), *P*<.001. For the proportion of those with “heparin,” slope=0.0013 (95% CI –0.00036 to 0.0030), *P*=.12.

Topics 3 and 7 both have cardiac catheterization for heart problems in common; for 5 out of 6 instances, the procedure or heparin administration was followed by hypotension (4 instances) that needed to be treated or heart rhythm deterioration (1 instance). To investigate whether these potential heparin AEs were increasing between July 2001 and June 2008, we plotted two measures of exposure (an invasive cardiac procedure code and “heparin”) and a measure of AE (“hypotension”). The proportion of admissions that had invasive cardiovascular procedure codes (Figure 7A,B) declined overall (Figure 7A), but had a local increase in period 3 compared to period 2. In contrast to the procedures, the words “heparin” and “hypotension” showed an overall rough increase over the entire time frame. We also noticed that the proportion of admissions with invasive cardiology codes that had the word “hypotension” increased gradually over time (Figures 7A,B), followed by a drop in the last quarter; the pattern was similar and weaker for the proportion of admissions with “heparin” that also had “hypotension.” There was a decrease in “hypotension” in the last quarter, both as a proportion of all admissions, and as a proportion of either indicator of having been exposed to heparin.

#### Other Common Topics for the Time-Based Case

**Figure 8:**
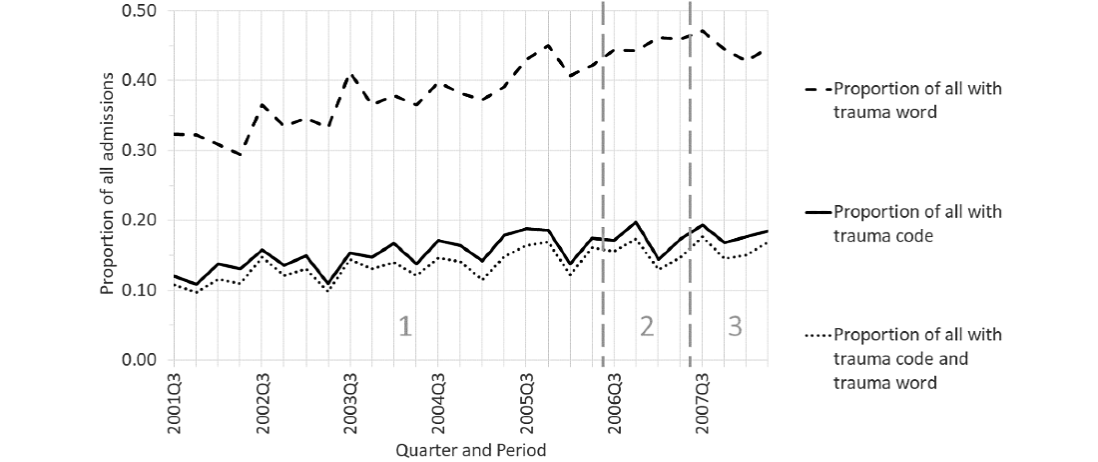
Trauma code, word, or both as a proportion of all admissions by quarter for the time-based case (see Table S4 [Multimedia Appendix 1] for search criteria details). For the proportion of trauma code, slope=0.0022 (95% CI 0.0014-0.0030), *P*<.001. For the proportion of the word “trauma,” slope=0.0057 (95% CI 0.0047-0.0067), *P*<.001. For the proportion with both trauma code and word, slope=0.0019 (95% CI 0.0012-0.0027), *P*<.001.

Topic 19 (and 13) corresponded with trauma. Figure 8 shows that trauma diagnosis and procedure codes increased steadily over time through periods 1 to 3.

**Figure 9:**
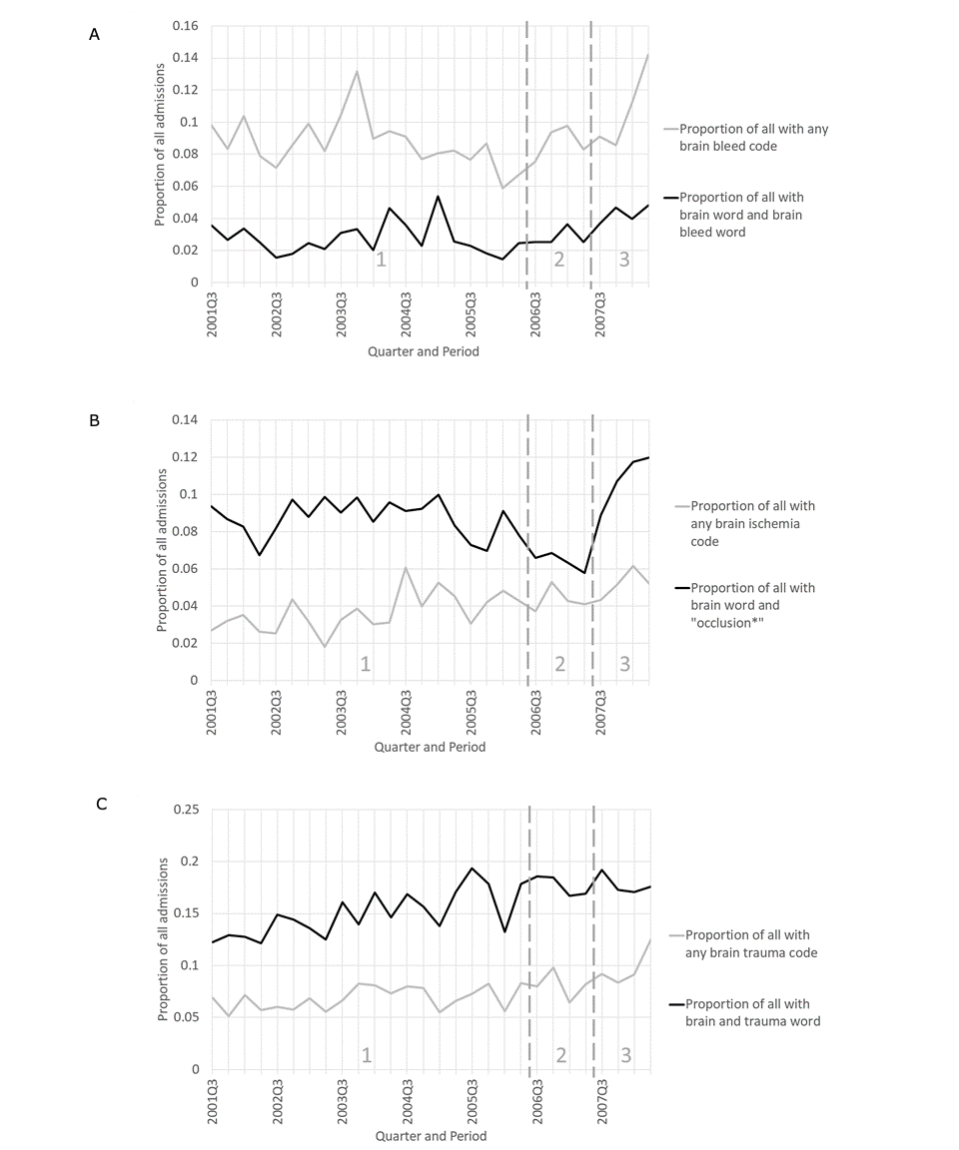
Brain ischemia codes or text words for (A) bleeding, (B) ischemia, and (C) trauma, as a proportion of all admissions by quarter for the time-based case (see Table S4 [Multimedia Appendix 1] for search criteria details). For brain bleed code, slope=0.00022 (95% CI –0.0006 to 0.0010), *P*=.61. For brain word and brain bleed word, slope=0.00039 (95% CI 0-0.00085), *P*=.10. For brain ischemia code, slope=0.00019 (95% CI 0.00051-0.0013), *P*<.001. For brain word and “occlusion*,” slope=0 (95% CI –0.00064 to 0.00080), *P*=.84. For brain trauma code, slope=0.0013 (95% CI 0.00073-0.0018), *P*<.001. For brain word and “trauma,” slope=0.0021 (95% CI 0.0014-0.0028), *P*<.001.

The brain topic (1 and 17, combined) was centered around admissions for brain injury (ie, bleeding, ischemia, or trauma). Figure 9A-C shows that there were local increases in codes for bleeding and ischemia for period 3 compared to period 2. There were slight increases in the codes for all three types of brain injuries overall. The text words indicating these conditions showed similar trends.

**Figure 10:**
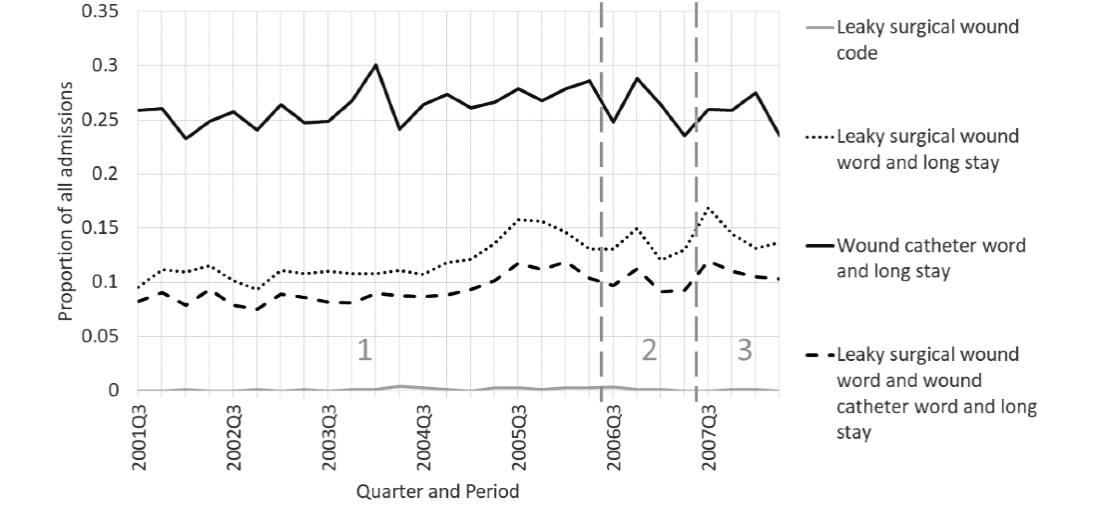
Excess draining from postsurgical wounds as a proportion of all admissions by quarter for the time-based case (see Table S4 [Multimedia Appendix 1] for search criteria details). For leaky surgical wound code, slope=0.000027 (95% CI –0.000028 to 0.000082), *P*=.34. For leaky surgical wound word and long stay, slope=0.0018 (95% CI 0.0012-0.0024), *P*<.001. For wound catheter word and long stay, slope=0.00038 (95% CI –0.00039 to 0.0012), *P*=.34. For leaky surgical wound word and wound catheter word and long stay, slope=0.0011 (95% CI 0.00071-0.0016), *P*<.001.

Topic 4 describes prolonged drainage after abdominal surgery. The index surgeries were performed before admission for 2 instances and during hospitalization for the third. Figure 10 shows that codes for wounds were quite infrequent. However, long patient stays with words for leaky surgical wound or catheter were more common, rose gradually over time, and had a local increase in period 3, compared to period 2.

Condition01 was the subject of the three admissions with the top match scores for topic 12. The codes and words were generally rare for the three periods and showed a local increase between periods 2 and 3.

#### Less Common Topics for the Time-Based Case

Summaries of admissions with topic matching scores for the less common topics are shown in Table S6 (Multimedia Appendix 1). We examined the top-scoring admissions matched to topic 11 and all admissions matched to the others. All admissions in this table had topic match scores for the index topic of <0.15 (column 2). Despite each admission in Table S6 (Multimedia Appendix 1) having at least one strong topic match score for at least one of the strong topics in Table S5 (Multimedia Appendix 1), the topics in Table S6 are distinct from those in Table S5. Some of the topics have admissions that have common aspects (topics 11, 10, 2, 9).

**Figure 11:**
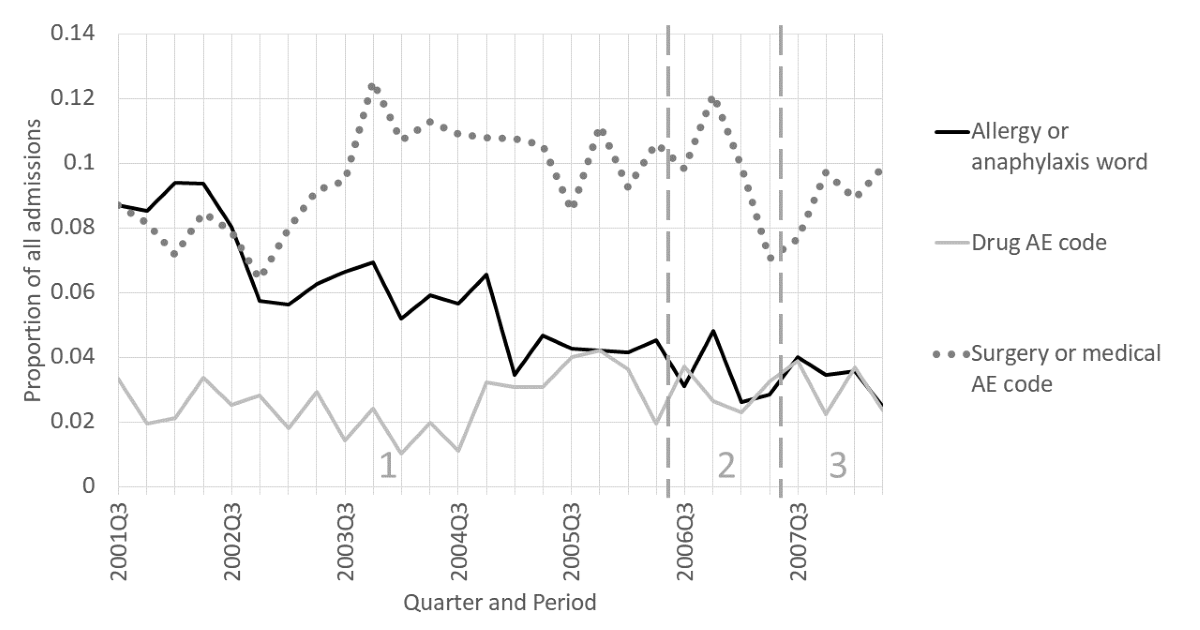
Allergy, anaphylaxis, and adverse effect (AE) as a proportion of admissions by quarter for the time-based case (see Table S4 [Multimedia Appendix 1] for search criteria details). For allergy or anaphylaxis word, slope=–0.0022 (95% CI –0.0027 to –0.0018), *P*<.001. For drug AE code, slope=0.00031 (95% CI –0.000079 to 0.00070), *P*=.12. For surgery or medical AE code, slope=0.00049 (95% CI –0.00022 to 0.0012), *P*=.18.

A total of 14 PAEs evident in the notes were distributed among the less common topics: 13 related to medical therapy (6 medications, 3 medical devices, 2 procedures, and 2 combinations) and 2 were nonmedical. Five drug and all of the medical device PAEs were published in the product labels and/or in the medical literature. Of the PAEs, 9 occurred outside the hospital and were related to the reason for admission. The diagnosis and procedure codes generally did not give enough information to understand the specific cause and associated PAE. Figure 11 shows that while the proportions over the 7 years of admissions with allergy and anaphylaxis words steadily decreased, the diagnosis codes for drug AEs and for surgical or procedure-related AEs increased slightly over time.

The other rare and infrequent terms, related diagnosis or procedure codes, and foreign language sentences were rare throughout all three time periods and increased during period 3.

### Discussion

We succeeded in our expectation of finding increases in clinical events and our hope of finding increases in PAEs, especially PAEs that were not attributed and thus likely not reported. We found increases in hypotension following heparin or presumed heparin exposure. Hypotension occurring in the cardiac catheterization lab could be a vasovagal reaction [95]. However, vasovagal reaction generally does not respond to fluids and drugs for raising blood pressure, and hypotension in all our observed patients did respond to treatment. Hypotension can occur as anaphylaxis begins and, alone, may reflect mild anaphylaxis. We note that the nurses and physicians that described the sequence of events did not link sudden hypotension to heparin and the diagnosis codes did not reflect any awareness of a link. The warnings from the FDA and the Centers for Disease Control and Prevention about heparin in the winter of 2007-2008 were for anaphylaxis due to contaminated heparin [96,97]. Knowledge of the extent of the distribution of contaminated heparin products was not specific, so it may have been in the hospital’s stock at the time. We had expected to see increases starting in 2006 because a few articles indicate heparin may have been adulterated before 2007 [98-100], but were surprised that the increases had started before 2006. The reduction in the last quarter coincided with recalls of contaminated heparin products and lend credibility to the idea that contaminated heparin was in slowly increasing use at this hospital for many years. It is surprising that such a high proportion of the invasive cardiac catheter patients in the last 2 years experienced hypotension following heparin exposure (either as explicitly documented administration or implicitly in the catheter coating).

Other types of clinical event changes we detected from periods 2 to 3 were increases in patients with common conditions (heart disease, brain injuries, trauma, and complex conditions associated with long hospital stays), increases in rare conditions, change in administration (foreign language portion), and PAEs of concern. The increases in common conditions may have reflected hospital marketing [101]. The increases in rare conditions could have reflected chance, or marketing as a referral center.

Nine of the PAEs happened outside the hospital and illustrate the utility of hospital records for monitoring severe reactions that occur in other health facilities or outside the health care system. Our method was useful for detecting words that are rare in hospital records, partly reflecting events that normally occur outside the hospital.

The topic with the highest document score exhibited behavior typical of a topic containing words that are common to most documents. The filter that was removing words comprised of only digits also removed digits from some words. This resulted in some high-frequency words entering the vocabulary. When topic modeling, this resulted in high scores for these common words in the topics where they were correlated (as expected, this happened in several topics) and created a common word topic (topic 18). This topic is a noise topic; the LDA model will put words that are low scoring and not correlated with other topics into their own noise topic in order to deal with noise and frequent words. Because this topic included words that were frequent in almost all documents, the document topic scores for this topic were high as expected [102]. This was dealt with by looking at the other more coherent topics that were assigned to each document (essentially ignoring this common-noise topic), capturing what most documents had in common. The top-scoring words in this topic that were general survived the ensemble filtering method as an artifact of the digit-removal step. For future work, we recommend removing this step from the filtering process and relying on the classification terms to filter out irrelevant variations of terms.

Our method worked despite:

- The known challenges posed by clinical text notes;
- Restriction to one major hospital;
- Lack of all surgical and non–critical care unit nursing notes, and variable lack of physician, nursing, or discharge summary notes, probably reflecting the hospital policy of gradually converting types of notes to EHRs [103];
- Errors up to several weeks in dates.

Different, and hopefully improved, results may be derived from EHR databases that are more complete and have actual dates.

## Discussion of the Shakespeare Method

### Comparison of the Shakespeare Method to Other Applications of LDA Topic Modeling

LDA topic modeling has been used for a variety of NLP tasks [63,64] (although it can also be used on other high-dimension data) such as text classification and filtering [65]. LDA topic modeling has been applied to the unstructured notes of EHRs to describe clinical groups [104-108] and predicting outcomes [109-116]. We were unable to find published instances of LDA topic-modeling applications for AE detection. Furthermore, we found none that apply LDA topic modeling to words or phrases in documents in the group of interest that are filtered to terms that most significantly distinguished a patient group of interest from a comparison group. This filtering process was essential for identifying topics describing the unique qualities of target versus comparison groups. Additionally, to our knowledge, we were the first to check the interpretation of documents with large numbers of topics with nontrivial scores.

The chosen number of topics was effective for identifying a range of PAEs. Evaluation of the overlap of topics and contents of documents identified for the varying numbers of topics has not been reported in the literature. Our iterative approach to evaluating different hyperparameters demonstrated, to our satisfaction, the relative stability of PAEs indicated by topics.

We determined the number of topics based on our experience of tuning the hyperparameters, the number of AEs reported in the literature, and the complexities of critical care patients. We were satisfied with the number because there was both overlap of topics that simultaneously had high word and document scores and some incoherent topics with low scores. As the number of topics becomes too large, additional topics are uninterpretable, and that as data set size increases, more robust topics are generated [117]. A systematic evaluation of the number of topics and other hyperparameters is always necessary for LDA topic modeling in a new setting.

LDA topic modeling has enabled identifying records for specific patients [118] who are or were clinically similar to an index patient. Identification of specific admissions is crucial to investigate PAEs. As reported in other studies [104], the topics with high scores tended to have good overlap of documents with similar clinical course and PAEs. Minor adjustments to the number of topics would still result in identifying the same PAE, even if different documents receive the top scores.

In the setting of using EHR notes with topic modeling to predict an outcome, studies noted that bigrams, trigrams, and unusual words added predictive ability [104,109]. Only unigrams survived our filtering process; however, different use cases or hyperparameter settings could yield useful multiword n-grams.

### Use of Classification to Filter Document Vectors

As noted before in the transfusion case, we were initially surprised that primarily unigrams (and not the longer sequences) appeared to play a significant role in distinguishing transfusion from comparison texts. We believe it is possible that enough unigrams that were part of meaningful phrases were also in other phrases or were significant on their own to result in relatively higher scores. For example, although “mechanical ventilation” conveys more meaning than just “mechanical” or “ventilation,” each word occurs singly or in phrases other than “mechanical ventilation.” We observed in the time-based case that similarly only unigrams survived classification.

Because bigrams and phrases were important in other LDA studies [104,109], we do not conclude that our unigram finding is necessarily applicable to other study settings. In this data set and blood transfusion and time-based cases, including only unigrams would not be expected to have changed the particular unigrams selected during the ensemble classification step. In other studies, it might be important to include n-grams where n>1.

Filtering the vectors to only terms that were important for focusing the topics on clinical conditions specific to the index condition, including reasons for and consequences of the condition, was important for identifying PAEs.

### Unsupervised Methods for the Surveillance of AEs in EHRs

We observed that the notes contained much more AE data than explicit discussion. We also found more AE data in the notes than in the diagnosis and procedure codes. Our prior analysis of diagnosis codes [57] demonstrated that in transfused versus nontransfused patients, there were some explicit TAEs, as well as more frequent diagnoses that were similar to TAEs (TRALI vs breathing difficulty, TACO vs acute kidney failure, etc). None of the documents we manually reviewed for this transfusion study bore any explicit TAE diagnosis code. Our prior and current analyses demonstrate that effective surveillance could benefit from using unstructured text as well as codes.

Our method was successful despite the limitations of this data set. The extent of records for each admission grew during the time that the data were collected because of the hospital’s policy of gradually adding more types of records to EHRs [103]. There was variation in the presence of nursing and physician progress notes in the examined records, which would not be present in the EHRs in systems that have long since become completely electronic. The presence of different types of records would logically have influenced the generated topics; for example, the topic on x-ray confirmation of device placement depends on the presence of radiology reports.

Much of our manual work to evaluate topics could be reduced with a combination of NLP and dictionaries of clinical terms. Dictionaries should include standard acronyms and common abbreviations, and should try to account for context when the meaning of a term could be ambiguous. The ability to decipher ongoing care notes will be important for noticing unrecognized signals of AEs.

## Conclusions

Topic analysis of statistically significant words in target documents found records indicative of PAEs, even if the clinician did not explicitly state an outcome was a suspected AE.

Among the PTAEs were unattributed evidence of TACO and TRALI. Some of the mid-2007 to mid-2008 PAEs were increased unattributed events consistent with heparin contamination–related AEs. Our results suggest that heparin contamination may have started before it was officially recognized in the winter of 2007-2008.

This method succeeded despite a wide variety of vocabulary (discipline-specific, context dependence, misspellings, multiple-word expressions, acronyms, personal abbreviations, etc) and formats (sentences, phrases, free lists, formatted lists, etc) used in the text. The Shakespeare method would likely generalize to other EHR notes and other types of medical texts. The computing tools are accessible and openly available. Their application to EHRs broadens the number of types of entities that could independently conduct surveillance of AEs.

It will be useful to adapt NLP methods to automate the abstraction of the notes; the tools will need to be tailored to the various formats used in the notes by different disciplines and individual clinicians. The expansion of vocabulary and acronym lists will also be useful. Automation tools will help to understand how PAEs are distributed within and among topics.

## Appendix

Multimedia Appendix 1 Supplementary tables.

## Abbreviations

AE: adverse event
C: comparison group
EHR: electronic health care record
FDA: Food and Drug Administration
LDA: latent Dirichlet allocation
LR: logistic regression
MIMIC-III: Medical Information Mart for Intensive Care III
NB: naïve Bayes
NLP: natural language processing
PAE: potential adverse event
PTAE: potential transfusion adverse event
T: transfusion group
TACO: transfusion-associated circulatory overload
TAE: transfusion adverse event
tPA: tissue plasminogen activator
TRALI: transfusion-related acute lung injury
