# Supplementary material for: Finding Potential Adverse Events in the Unstructured Text of Electronic Health Care Records: Development of the Shakespeare Method
Source: JMIRx Med. 2021 Aug 11;2(3):e27017. doi: 10.2196/27017 (PMC10414364; doi:10.2196/27017)
Supplement: Multimedia Appendix 1 [file xmed_v2i3e27017_app1.docx]

**Multimedia Appendix 1:** Supplementary tables.

Table S1. For each topic in the transfusion case, score for the top term, top 20 term, top document score, and the distribution of documents by document score range.

| Topic | | | Top document score | # documents in topic score range | | | | |
| --- | --- | --- | --- | --- | --- | --- | --- | --- |
| Topic # | Top term score | Top 20 terms |  | >0.03 | >0.5 | >0.2 to <0.5 | >0.1 to <0.2 | >0.03 to <0.1 |
| 17 | 20655 | avr, tee, atheroma, mvr, sternotomy, incisions, cvicu, sec, appendage, prosthesis, regulations, hcfa, certify, temporary, laa, svg, pounds, velocity, swan, bioprosthetic | 0.994 | 5396 | 1038 | 1456 | 997 | 1905 |
| 7 | 13074 | epidural, fusion, l3, l2, thoracotomy, hardware, t12, laminectomy, brace, t11, c2, t3, t10, t7, t9, t8, paraspinal, t6, ortho, t5 | 0.984 | 2133 | 148 | 332 | 415 | 1238 |
| 3 | 9447 | femur, orif, ortho, comminuted, injuries, collar, mva, tsicu, fixation, pubic, elbow, filter, lac, tibia, tibial, humerus, struck, tib, reduction, fragment | 0.983 | 3099 | 363 | 852 | 644 | 1240 |
| 35 | 24272 | ci, iabp, swan, svo2, milrinone, csru, amiodarone, ganz, ntg, paced, svg, amio, balloon, fick, sternotomy, cco, cdi, prbc, lima, pac | 0.971 | 6399 | 1398 | 2176 | 984 | 1841 |
| 42 | 10586 | dopamine, mso4, swan, amiodarone, captopril, paced, dopa, eve, haldol, conts, maps, pvcs, wedge, nph, pad, ntg, svr, ganz, careview, 1st | 0.926 | 12983 | 594 | 3626 | 3933 | 4830 |
| 18 | 12454 | egd, colonoscopy, gib, prbc, melena, endoscopy, brbpr, prbcs, scope, transfused, transfusion, gastritis, diverticulosis, bright, lavage, hcts, prep, clots, maroon, transfusions | 0.925 | 4532 | 534 | 1261 | 822 | 1915 |
| 24 | 12191 | needle, guid, vena, ir, cava, guidewire, filter, prepped, draped, exchanged, peel, table, micropuncture, brachial, flushed, supervising, secured, placment, double, basilic | 0.909 | 4503 | 30 | 634 | 1349 | 2490 |
| 44 | 11474 | icp, pupil, evacuation, nipride, tragus, withdraws, angio, lue, rue, nailbed, opening, noxious, 4mm, briskly, reflex, corneal, sluggish, cpp, stimulation, sw | 0.882 | 2980 | 54 | 861 | 652 | 1413 |
| 11 | 6953 | aaa, iliac, fem, pvd, tibial, rle, lle, dopplerable, thrombectomy, saphenous, endovascular, vicu, cca, duplex, pedis, infrarenal, fasciotomy, sfa, brachial, sec | 0.878 | 3330 | 104 | 457 | 681 | 2088 |
| 1 | 6242 | ends, dobbhoff, multifocal, dobhoff, cavoatrial, projects, projecting, airspace, devices, brachiocephalic, nodular, heterogeneous, newly, defined, superimposed, compressibility, pole, aeration, pls, cava | 0.875 | 11183 | 149 | 2066 | 3036 | 5932 |
| 30 | 8324 | lymphoma, chemo, chemotherapy, marrow, aml, acyclovir, neutropenia, bmt, neutropenic, onc, myeloma, leukemia, plts, thrombocytopenia, cefepime, platelet, fungal, pancytopenia, gvhd, cmv | 0.860 | 2254 | 110 | 510 | 445 | 1189 |
| 9 | 15046 | residuals, vented, mdi, fent, copious, orally, mushroom, sxn, abp, tlc, overnoc, conts, haldol, 30cc, lip, los, 50cc, promote, 50mcg, 10cc | 0.856 | 8672 | 282 | 1980 | 2289 | 4121 |
| 6 | 30540 | dialysis, cvvh, crrt, hemodialysis, cvvhd, tunneled, anuric, arf, levophed, cvvhdf, filter, temporary, running, quinton, vasopressin, ir, hugger, tunnel, clotted, bair | 0.852 | 2182 | 1 | 164 | 757 | 1260 |
| 13 | 9976 | tpn, lap, ileostomy, colectomy, colostomy, ostomy, stoma, ex, jp, laparotomy, exploratory, perforation, anastomosis, perforated, staples, loop, pouch, appliance, adhesions, transverse | 0.814 | 2825 | 35 | 539 | 829 | 1422 |
| 22 | 11163 | prbc, ffp, transfusion, arf, transfuse, transfused, prbcs, nstemi, thrombocytopenia, coagulopathy, dic, vit, coags, resuscitation, guaiac, goals, fibrinogen, cri, plts, hemolysis | 0.813 | 10751 | 127 | 2185 | 3184 | 5255 |
| 12 | 25307 | cirrhosis, portal, lactulose, paracentesis, tips, encephalopathy, transplant, hcv, alcoholic, ffp, varices, esld, icteric, hepatology, tap, splenomegaly, rifaximin, jaundice, organ, hepatorenal | 0.778 | 2668 | 85 | 888 | 681 | 1014 |
| 37 | 29924 | transplant, embolization, jp, extravasation, pel, angio, tacrolimus, olt, selective, transplanted, duplex, ea, celiac, waveforms, embo, portal, resistive, addl, needle, coils | 0.765 | 2064 | 38 | 635 | 524 | 867 |
| 14 | 2962 | labetolol, ovarian, vaginal, hysterectomy, uterus, uterine, gyn, ob, frozen, plasma, fibroids, fibroid, fresh, tomography, packed, debulking, oophorectomy, computed, peritoneal, pad | 0.699 | 1140 | 17 | 113 | 138 | 872 |
| 20 | 13107 | pancreatic, tpn, bile, duct, pigtail, cholecystitis, intrahepatic, collections, ductal, percutaneous, bilious, portal, guided, sludge, pseudocyst, ducts, cholangiogram, tail, splenic, jaundice | 0.685 | 3296 | 59 | 572 | 741 | 1924 |
| 2 | 10078 | esophageal, varices, egd, hematemesis, octreotide, variceal, endoscopy, banding, ugib, coffee, nadolol, esophagitis, melena, tear, gastropathy, gib, sucralfate, portal, banded, prbc | 0.670 | 1518 | 16 | 290 | 374 | 838 |
| 10 | 12074 | bilious, simv, sxn, jp, staples, serous, serosang, specifics, abgs, dsd, gravity, softly, drg, drng, sang, spont, impact, acceptable, pp, ips | 0.670 | 7465 | 47 | 1745 | 2275 | 3398 |
| 40 | 5248 | pseudoaneurysm, hematuria, nephrostomy, flank, nephrectomy, extravasation, rp, iliac, splenic, thigh, clots, ureteral, pole, stranding, rupture, ureter, prbc, cbi, irrigation, psoas | 0.661 | 2673 | 6 | 315 | 565 | 1787 |
| 33 | 8714 | thoracentesis, colitis, tamponade, loculated, hemothorax, tap, argatroban, sized, hydropneumothorax, drained, ulcerative, hit, amiodarone, reaccumulation, tapped, uc, focused, evacuation, serosanguinous, cdiff | 0.642 | 1342 | 1 | 52 | 187 | 1102 |
| 26 | 27305 | cmh2o, iu, mmol, ve, pao2, pip, consistency, gluconate, sbt, mono, consults, wgt, pedis, sit, fibrinogen, cmv, subjective, teeth, phenylephrine, piperacillin | 0.634 | 3097 | 17 | 1263 | 862 | 955 |
| 25 | 91911 | trach, peg, tracheostomy, collar, trached, words, mdi, copious, mouthing, portex, residuals, tm, muir, trache, pmv, trials, nph, gastrostomy, sxn, bronch | 0.631 | 2356 | 1 | 451 | 877 | 1027 |
| 41 | 20440 | coccyx, duoderm, serous, buttocks, tears, dsd, anasarca, weeping, edematous, residuals, barrier, reddened, oozing, heel, mushroom, sites, sediment, scrotum, powder, excoriated | 0.612 | 6674 | 5 | 780 | 1938 | 3951 |
| 4 | 12870 | vac, closure, debridement, flap, packing, plastics, washout, thigh, jp, plastic, paralyzed, compartment, dressings, packed, twitches, tof, exploration, necrotizing, serosang, serous | 0.606 | 1864 | 1 | 157 | 331 | 1375 |
| 23 | 13137 | fistula, pea, splenectomy, amputation, bka, stump, pvd, aka, gangrene, methadone, necrotic, peritoneal, revision, thrill, amp, enterocutaneous, gangrenous, tma, embolectomy, dextrose | 0.598 | 1559 | 3 | 42 | 238 | 1276 |
| 19 | 6256 | pseudomonas, staph, meropenem, aureus, cefepime, resistant, tobramycin, empyema, coag, ceftazidime, staphylococcus, oxacillin, klebsiella, piperacillin, rifampin, methicillin, cocci, tobra, rod, mssa | 0.553 | 1312 | 1 | 28 | 183 | 1100 |
| 32 | 2157 | sternotomy, snds, median, bbs, pej, derm, glucoses, req, nph, fld, aao, fsg, slt, periph, intermit, brth, epoetin, occas, epogen, liqs | 0.506 | 707 | 1 | 1 | 61 | 644 |
| 29 | 19219 | ards, tpn, pao2, pcv, bal, fent, levophed, multifocal, alveolar, paralyzed, midazolam, bronch, abgs, pip, cmv, cooling, balloon, gtts, airspace, vasopressin | 0.485 | 1763 | 0 | 54 | 322 | 1387 |
| 39 | 19348 | levophed, septic, bacteremia, vegetation, linezolid, tee, meropenem, maps, vasopressin, vre, vegetations, pressor, cardiogenic, dopamine, gnr, mssa, daptomycin, sacral, gent, growing | 0.406 | 3297 | 0 | 158 | 752 | 2387 |
| 34 | 8780 | bronch, hemithorax, pneumonectomy, cfa, adenoca, cesarean, shifted, hypogastric, iib, filled, accreta, lsfa, stump, inflation, ctx2, tring, plummeted, amniotic, coverge, enourage | 0.328 | 199 | 0 | 8 | 25 | 166 |
| 38 | 9958 | ileus, kub, distension, colonic, filled, erect, cecum, pneumatosis, intraperitoneal, transverse, decubitus, cecal, decompression, gaseous, fecal, colitis, pseudo, guillain, ppn, cdiff | 0.309 | 617 | 0 | 4 | 43 | 570 |
| 5 | 7772 | pyloric, duodenum, duodenal, gj, bulb, pylorus, gastrojejunostomy, antrum, jejunal, outlet, injected, ugi, beyond, jejunostomy, coffee, eus, dermatomyositis, antrectomy, du, pylori | 0.274 | 682 | 0 | 6 | 71 | 605 |
| 43 | 633 | colloid, chnage, reop, h3, enabled, hypotenstion, dextrocardia, hfov, hindi, debranching, dssgs, cytogam, pneumthorax, dimininshed, preps, pao, vits, coagulaopathy, 30yr, 84cc | 0.248 | 8 | 0 | 1 | 1 | 6 |
| 15 | 1854 | aids, fiance, burkitt, tonsil, pml, burkitts, ivac, cmv, regularized, pulmonay, fenoldopam, hj, 0400am, ntt, corgard, hypothalamus, intrabd, dauboff, interruptions, plateletts | 0.157 | 72 | 0 | 0 | 4 | 68 |
| 28 | 2292 | gluteal, apml, atra, gluteus, biphenotypic, maximus, medius, dysfuction, paraganglioma, supracoronary, basilic, cd13, promyelocytic, exfoliative, fusarium, presbyterian, cd11c, lsi, swap, lidex | 0.152 | 47 | 0 | 0 | 5 | 42 |
| 0 | 6254 | seal, crepitus, vats, pneumomediastinum, 20cm, perforation, waterseal, lucency, pneumopericardium, esophageal, perf, micronodular, indian, accelerating, 110bpm, cortstim, coccidiomycosis, pneumothrax, amphoteracin, 54yr | 0.148 | 312 | 0 | 0 | 13 | 299 |
| 21 | 652 | intraoperative, trisegmentectomy, germ, doxacurium, wkness, __________, pulomonary, stenotrophamonas, ante, thearphy, 19cc, moderatly, abe, dital, uncomfortably, indapamide, tothe, doxicurium, bottocks, centigrade | 0.124 | 32 | 0 | 0 | 2 | 30 |
| 36 | 78 | cimetidine, 67yom, repacking, rectopexy, ridden, wetness, q72hr, thyroidal, incompetence, 250g, ketalorac, gaping, deoderm, bilingual, retroperitonal, msse, telem, cinemet, chondritis, ifed8f4k | 0.085 | 5 | 0 | 0 | 0 | 5 |
| 16 | 1573 | oxide, nitric, bigeminal, incompatible, foscarnet, ino, redundant, 40ppm, uncorrected, beriberi, pose, retrohepatic, 20ppm, percutanous, physio, plam, no2, 10ppm, withstand, compatibility | 0.085 | 27 | 0 | 0 | 0 | 27 |
| 31 | 26 | vulvectomy, sqcca, nias, noncemented, cachexic, cholesteatoma, vwd, shuffled, nonstd, contagious, mycardial, cervival, goven, norfloxacin, dobloff, remov, hyperdyanmic, decsions, garbling, hemacrit | 0.080 | 3 | 0 | 0 | 0 | 3 |
| 8 | 322 | deaf, cmml, cto, 83y, 18m, t103, postponement, anakinra, myelomonocytic, rsfa, suspend, fliter, serousanguous, initail, glob, intrathroacic, emolization, eighties, breakdwon, courseness | 0.057 | 13 | 0 | 0 | 0 | 13 |
| 27 | 100 | 32mm, fulguration, 54yom, day9, cret, monitro, inactivated, iwht, 8uffp, addendem, day10, cleasned, hypermagnesemia, stenotomy, lings, cc7c, multag, urcx, emer, bjork | 0.043 | 5 | 0 | 0 | 0 | 5 |

Table S2. Topic interpretations in the transfusion case based on top 3 scoring documents in each topic and randomly selected documents. We present the 81 documents with T date information and without an obvious alternate explanation for the AE. Documents are the unit of analysis. Topics are grouped by similarity of interpretation (some did not have a coherent interpretation); the cardiovascular, lung failure, and volume overload topic groups are also grouped into a super PTAE topic group. “Codes” refers to billing codes. ARDS: acute respiratory distress syndrome. CABG: coronary artery bypass graft. GI: gastrointestinal. HIV: human immunodeficiency virus. MVA: motor vehicle accident. tPA: tissue plasminogen activator.

| TOPIC GROUP, TOPIC SUBGROUP, or Topic number  (45-) and topic interpretation | # documents | Presence of notes | | | AE attribu-tions | | | Heart AE after T | | | | | Lung AE  after T | | | | Volume AE after T | | | Noninfection fever /chills after T | PTAE summaries | | | | T dates in notes | T presumed during surgery |
| --- | --- | --- | --- | --- | --- | --- | --- | --- | --- | --- | --- | --- | --- | --- | --- | --- | --- | --- | --- | --- | --- | --- | --- | --- | --- | --- |
|  |  | Physician progress | Nurse progress | Discharge summary | AE attributed to T in notes | AE attributed to T in codes | Codes mention T | Atrial fibrillation | Tachycardia | Bradycardia | Other heart rhythm abnormality | Hypotension | Hypoxia | Mechanical ventilation | Bilateral Pleural Effusion | Pulmonary edema | Edema | Acute renal failure | Diuresis |  | Heart | Lung | Volume | Any PTAE |  |  |
| PTAE TOPIC | 29 | 6 | 21 | 26 |  |  | 2 | 2 | 3 | 1 | 2 | 8 | 4 | 6 | 7 | 5 | 3 | 2 | 2 | 4 | 12 | 14 | 5 | 18 | 25 | 4 |
|  | | | | | | | | | | | | | | | | | | | | | | | | | | |
| CARDIO-VASCULAR | 9 |  | 4 | 8 |  |  |  | 2 | 1 | 1 | 1 | 4 |  | 1 | 1 |  |  |  | 1 | 1 | 6 | 3 | 1 | 6 | 6 | 3 |
| 17 heart valve repair | 3 |  |  | 3 |  |  |  | 1 |  |  |  |  |  |  | 1 |  |  |  |  |  | 1 | 1 |  | 1 | 2 | 1 |
| 33 tapped pericardial effusion | 1 |  |  | 1 |  |  |  |  |  |  | 1 | 1 |  | 1 |  |  |  |  |  |  | 1 | 1 |  | 1 | 1 |  |
| 35 CABG | 2 |  | 2 | 2 |  |  |  | 1 |  |  |  | 1 |  |  |  |  |  |  | 1 | 1 | 2 |  | 1 | 2 | 1 | 1 |
| 42 heart attack | 2 |  | 2 | 1 |  |  |  |  | 1 | 1 |  | 1 |  |  |  |  |  |  |  |  | 1 |  |  | 1 | 1 | 1 |
| 11 vascular repair | 1 |  |  | 1 |  |  |  |  |  |  |  | 1 |  | 1 |  |  |  |  |  |  | 1 | 1 |  | 1 | 1 |  |
|  | | | | | | | | | | | | | | | | | | | | | | | | | | |
| LUNG FAILURE | 14 | 5 | 13 | 13 |  |  | 2 |  | 2 |  | 1 | 3 | 4 | 5 | 4 | 4 | 3 | 1 | 1 | 3 | 5 | 8 | 3 | 8 | 14 |  |
| 9, 10 mechanical ventilation | 6 |  | 6 | 6 |  |  | 2 |  | 1 |  |  | 1 | 2 | 2 | 1 | 1 | 2 |  |  | 2 | 1 | 2 | 1 | 2 | 6 |  |
| 26 mechanical ventilation/ fall trauma | 3 | 3 | 3 | 2 |  |  |  |  |  |  |  |  |  | 1 | 1 | 1 |  |  |  |  |  | 2 |  | 2 | 3 |  |
| 29 ARDS | 2 | 1 | 2 | 2 |  |  |  |  |  |  |  | 1 |  | 1 |  | 1 | 1 | 1 |  | 1 | 1 | 2 | 1 | 2 | 2 |  |
| 16 nitric oxide therapy/ ? | 3 | 1 | 2 | 3 |  |  |  |  | 1 |  | 1 | 1 | 2 | 1 | 2 | 1 |  |  | 1 |  | 3 | 2 | 1 | 3 | 3 |  |
|  |  |  |  |  |  |  |  |  |  |  |  |  |  |  |  |  |  |  |  |  |  |  |  |  |  |  |
| VOLUME OVERLOAD | 6 | 1 | 4 | 5 |  |  |  |  |  |  |  | 1 |  |  | 2 | 1 |  | 1 |  |  | 1 | 3 | 1 | 4 | 5 | 1 |
| 37 kidney failure | 3 | 1 | 1 | 3 |  |  |  |  |  |  |  | 1 |  |  |  |  |  | 1 |  |  | 1 |  | 1 | 1 | 2 | 1 |
| 41 edema/ skin breakdown | 3 |  | 3 | 2 |  |  |  |  |  |  |  |  |  |  | 2 | 1 |  |  |  |  |  | 3 |  | 3 | 3 |  |
|  |  |  |  |  |  |  |  |  |  |  |  |  |  |  |  |  |  |  |  |  |  |  |  |  |  |  |
| REASON TO TRANSFUSE TOPIC | 42 | 5 | 20 | 40 | 1 |  | 5 | 3 | 6 | 2 | 1 | 9 | 4 | 6 | 7 | 3 |  | 1 | 3 | 6 | 16 | 10 | 3 | 20 | 34 | 7 |
|  | | | | | | | | | | | | | | | | | | | | | | | | | | |
| BLOOD | 9 | 2 | 5 | 9 | 1 |  |  |  |  |  |  | 2 | 1 | 2 |  | 1 |  |  | 1 | 2 | 2 | 2 | 1 | 3 | 9 |  |
| 24 TPA to lyse thrombus | 1 |  | 1 | 1 |  |  |  |  |  |  |  | 1 |  |  |  |  |  |  |  | 1 | 1 |  |  | 1 | 1 |  |
| 30 blood disease | 2 |  |  | 2 | 1 |  |  |  |  |  |  | 1 | 1 | 1 |  | 1 |  |  | 1 | 1 | 1 | 1 | 1 | 1 | 2 |  |
| 22 anemia | 1 | 1 | 1 | 1 |  |  |  |  |  |  |  |  |  |  |  |  |  |  |  |  |  |  |  |  | 1 |  |
| 20 pancreas; liver | 2 |  | 1 | 2 |  |  |  |  |  |  |  |  |  | 1 |  |  |  |  |  |  |  | 1 |  | 1 | 2 |  |
| 2 esophageal varices banding | 3 | 1 | 2 | 3 |  |  |  |  |  |  |  |  |  |  |  |  |  |  |  |  |  |  |  |  | 3 |  |
|  | | | | | | | | | | | | | | | | | | | | | | | | | | |
| 34 lung cancer | 3 |  | 1 | 3 |  |  | 1 | 1 | 1 |  |  |  | 1 |  | 2 |  |  |  |  |  | 1 | 2 |  | 2 | 3 |  |
|  | | | | | | | | | | | | | | | | | | | | | | | | | | |
| GI | 12 |  | 4 | 11 |  |  | 1 | 1 | 2 |  | 1 | 2 | 1 | 3 | 3 | 1 |  |  | 1 |  | 5 | 2 |  | 3 | 6 | 5 |
| 18 GI bleeding | 1 |  |  | 1 |  |  |  |  |  |  |  |  |  |  |  |  |  |  |  |  |  |  |  |  | 1 |  |
| 13 colonostomy | 3 |  |  | 3 |  |  |  |  | 1 |  |  | 1 |  | 2 | 1 |  |  |  | 1 |  | 2 |  |  |  |  | 3 |
| 38 colon problem/ ? | 3 |  | 1 | 3 |  |  | 1 |  | 1 |  | 1 |  | 1 |  | 1 | 1 |  |  |  |  | 2 | 1 |  | 2 | 2 | 1 |
| 5 GI | 3 |  | 2 | 3 |  |  |  |  |  |  |  |  |  |  |  |  |  |  |  |  |  |  |  |  | 2 |  |
| 0 esophagus/ ? | 2 |  | 1 | 1 |  |  |  | 1 |  |  |  | 1 |  | 1 | 1 |  |  |  |  |  | 1 | 1 |  | 1 | 1 | 1 |
|  |  |  |  |  |  |  |  |  |  |  |  |  |  |  |  |  |  |  |  |  |  |  |  |  |  |  |
| INFECTION | 6 | 3 | 5 | 6 |  |  | 2 | 1 | 1 | 1 |  | 3 |  |  |  | 1 |  | 1 | 1 | 1 | 4 | 1 | 2 | 5 | 6 |  |
| 23 below knee amputation | 3 | 1 | 2 | 3 |  |  | 1 |  |  | 1 |  | 2 |  |  |  |  |  |  | 1 |  | 2 |  | 1 | 3 | 3 |  |
| 39 infection | 2 | 2 | 2 | 2 |  |  |  | 1 |  |  |  | 1 |  |  |  |  |  |  |  |  | 1 |  |  | 1 | 2 |  |
| 15 HIV | 1 |  | 1 | 1 |  |  | 1 |  | 1 |  |  |  |  |  |  | 1 |  | 1 |  | 1 | 1 | 1 | 1 | 1 | 1 |  |
|  | | | | | | | | | | | | | | | | | | | | | | | | | | |
| OTHER SURGERY | 12 |  | 5 | 11 |  |  | 1 |  | 2 | 1 |  | 2 | 1 | 1 | 2 |  |  |  |  | 3 | 4 | 3 |  | 7 | 10 | 2 |
| 7 spine surgery | 2 |  |  | 2 |  |  |  |  |  |  |  |  |  |  |  |  |  |  |  |  |  |  |  |  | 1 | 1 |
| 3 bone trauma from MVA | 2 |  | 1 | 2 |  |  |  |  |  |  |  | 1 |  |  |  |  |  |  |  | 1 | 1 |  |  | 2 | 2 |  |
| 14 uterus surgery | 3 |  | 1 | 3 |  |  |  |  | 1 |  |  |  |  |  |  |  |  |  |  | 1 | 1 |  |  | 1 | 3 |  |
| 40 retroperitoneal bleed/ bleeding in bladder | 3 |  | 2 | 2 |  |  | 1 |  |  | 1 |  |  | 1 |  | 1 |  |  |  |  |  | 1 | 2 |  | 2 | 3 |  |
| 4 plastic surgery flap wound care | 2 |  | 1 | 2 |  |  |  |  | 1 |  |  | 1 |  | 1 | 1 |  |  |  |  | 1 | 1 | 1 |  | 2 | 1 | 1 |
|  | | | | | | | | | | | | | | | | | | | | | | | | | | |
| OTHER TOPICS | 10 | 4 | 6 | 11 |  |  | 1 |  |  |  |  | 2 | 2 | 2 | 1 |  |  |  |  | 1 | 2 | 4 |  | 6 | 9 | 1 |
|  | | | | | | | | | | | | | | | | | | | | | | | | | | |
| ILL-DEFINED | 10 | 4 | 6 | 11 |  |  | 1 |  |  |  |  | 2 | 2 | 2 | 1 |  |  |  |  | 1 | 2 | 4 |  | 6 | 9 | 1 |
| 32 | 1 |  | 1 | 2 |  |  |  |  |  |  |  |  | 1 |  |  |  |  |  |  |  |  | 1 |  | 1 | 1 |  |
| 43 | 1 | 1 | 1 | 1 |  |  |  |  |  |  |  |  |  |  |  |  |  |  |  |  |  |  |  |  | 1 |  |
| 28 | 1 |  |  | 1 |  |  |  |  |  |  |  |  |  |  | 1 |  |  |  |  | 1 | 1 | 1 |  | 2 | 1 |  |
| 21 | 1 |  |  |  |  |  |  |  |  |  |  | 1 |  | 1 |  |  |  |  |  |  |  |  |  |  |  | 1 |
| 36 | 2 | 2 | 2 | 3 |  |  | 1 |  |  |  |  | 1 | 1 |  |  |  |  |  |  |  | 1 | 1 |  | 2 | 2 |  |
| 31 | 2 |  |  | 2 |  |  |  |  |  |  |  |  |  |  |  |  |  |  |  |  |  |  |  |  | 2 |  |
| 8 | 1 |  | 1 | 1 |  |  |  |  |  |  |  |  |  |  |  |  |  |  |  |  |  |  |  |  | 1 |  |
| 27 | 1 | 1 | 1 | 1 |  |  |  |  |  |  |  |  |  | 1 |  |  |  |  |  |  |  | 1 |  | 1 | 1 |  |
|  | | | | | | | | | | | | | | | | | | | | | | | | | | |
| RANDOM TOTAL | 20 | 1 | 15 | 19 | 2 |  | 5 |  | 3 | 1 |  | 2 | 2 | 1 | 3 |  | 5 | 1 | 5 |  | 4 | 4 | 7 | 12 | 15 | 3 |

Table S3. Topic interpretations based on the three top-scoring documents with 13 or 14 topics. “Codes” refers to billing codes. NA: not applicable.

| Group | # documents | Presence of notes | | | AE attribu-tions | | | Heart AE after T | | | | | Lung AE after T | | | | Volume AE after T | | | Noninfection fever /chills after T | PTAE summaries | | | | T dates in notes | T presumed during surgery | Remarks |
| --- | --- | --- | --- | --- | --- | --- | --- | --- | --- | --- | --- | --- | --- | --- | --- | --- | --- | --- | --- | --- | --- | --- | --- | --- | --- | --- | --- |
|  |  | Physician progress | Nurse progress | Discharge summary | AE attributed to T in notes | AE attributed to T in codes | Codes mention T | Atrial fibrillation | Tachycardia | Bradycardia | Other heart rhythm abnormality | Hypotension | Hypoxia | Mechanical ventilation | Bilateral Pleural Effusion | Pulmonary edema | Edema | Acute renal failure | Diuresis |  | Heart | Lung | Volume | Any PTAE |  |  |  |
| TOTAL 13 or 14 topics | 8 | 3 | 8 | 8 | 2 |  | 2 | 1 | 1 | 1 | 1 | 1 | 1 |  | 3 | 1 | 1 | 2 |  |  | 1 | 5 | 2 | 5 | 8 |  |  |
|  | | | | | | | | | | | | | | | | | | | | | | | | | | | |
| WITHOUT ADVANCED CANCER | 5 | 1 | 5 | 5 | 2 |  |  | 1 | 1 | 1 | 1 | 1 | 1 |  | 2 |  | 1 | 2 |  |  | 1 | 3 | 2 | 3 | 5 |  |  |
| 13 or 14 topics without advanced cancer | 1 | 1 | 1 | 1 |  |  |  |  |  |  |  |  |  |  |  |  |  |  |  |  |  |  |  |  | 1 |  |  |
|  | 1 |  | 1 | 1 | 1 |  |  |  |  |  |  |  |  |  |  |  |  |  |  |  |  |  |  |  | 1 |  | Thrombo-cytopenia attributed to heparin. |
|  | 1 |  | 1 | 1 | 1 |  |  | 1 | 1 | 1 | 1 | 1 |  |  | 1 |  |  | 1 |  |  | 1 | 1 | 1 | 1 | 1 |  | Notes cite delayed transfusion reaction. |
|  | 1 |  | 1 | 1 |  |  |  |  |  |  |  |  |  |  | 1 |  |  |  |  |  |  | 1 |  | 1 | 1 |  |  |
|  | 1 |  | 1 | 1 |  |  |  |  |  |  |  |  | 1 |  |  |  | 1 | 1 |  |  |  | 1 | 1 | 1 | 1 |  | Worsening kidneys attributed to Tobramycin rather than transfusion. Also had new congestive heart failure |
|  | | | | | | | | | | | | | | | | | | | | | | | | | | | |
| WITH ADVANCED CANCER | 3 | 2 | 3 | 3 |  |  | 2 |  |  |  |  |  |  |  | 1 | 1 |  |  |  |  |  | 2 |  | 2 | 3 |  |  |
| 13 or 14 topics with advanced cancer | 1 |  | 1 | 1 |  | NA | NA |  |  |  |  |  |  |  |  |  |  |  |  |  |  |  |  |  | 1 |  | Advanced cancer. |
|  | 1 | 1 | 1 | 1 |  |  | 1 |  |  |  |  |  |  |  | 1 |  |  |  |  |  |  | 1 |  | 1 | 1 |  | Advanced cancer |
|  | 1 | 1 | 1 | 1 |  |  | 1 |  |  |  |  |  |  |  |  | 1 |  |  |  |  |  | 1 |  | 1 | 1 |  | Thrombi. Liver cancer. |

Table S4. Search criteria used to analyze periods 1, 2, and 3 in the time-based case.

| Concept | Search criteria (any, unless otherwise specified) | Used in figure |
| --- | --- | --- |
| Invasive cardiovascular procedure code | - 3891 Arterial catheterization - 3961 Extracorporeal circulation auxiliary to open heart surgery - 3965 Extracorporeal membrane oxygenation [ECMO] - 3966 Percutaneous cardiopulmonary bypass | 7 |
| Heparin word | “heparin” | 7 |
| Hypotension word | “hypotension” | 7 |
| Trauma diagnosis code | - 800* to 829* [Fracture] - 830* to 869* [Dislocations, sprains, strains, and internal injury of cranium, chest, abdomen, pelvis] - 870* to 897* [Open wound] - 900* to 904* Injury to blood vessels - 905* Late effects of musculoskeletal and connective tissue injuries - 9060 to 9064 [Late effects of open wound, superficial injury, contusion, or crushing] - 907* Late effects of injuries to the nervous system - 908* Late effects of other and unspecified injuries - 910* to 924* [Superficial injury and contusion with intact skin surface] - 925* to 929* Crushing injury - 950* to 957* Injury To Nerves And Spinal Cord - 958* to 959* Certain Traumatic Complications And Unspecified Injuries | 8 |
| Trauma procedure code | - 7670 to 7679 [reduction of fracture] - 7810 to 7819 [application of fixator device] - 7900 to 7939 [reduction of fracture] - 7960 to 7969 [debridement of open fracture site] - 7990 to 7999[operation on bone injury] | 8 |
| Trauma word | - “contusion” - “fall” - “fracture” - “mva” - “mvc” - “trauma” | 8 |
| Brain bleed diagnosis code | - 3481* Anoxic brain damage - 3484* Compression of brain - 430* Subarachnoid hemorrhage - 431 Subarachnoid hemorrhage - 432 Other and unspecified intracranial hemorrhage - 8042* Closed fractures involving skull or face with other bones with subarachnoid subdural and extradural hemorrhage - 8043* Closed fractures involving skull or face with other bones, with other and unspecified intracranial hemorrhage - 8047* Open fractures involving skull or face with other bones with subarachnoid subdural and extradural hemorrhage - 8048* Open fractures involving skull or face with other bones with other and unspecified intracranial hemorrhage - 852** Subarachnoid subdural and extradural hemorrhage following injury - 853** Therapeutic and unspecified intracranial hemorrhage following injury | 9 |
| Brain bleed procedure code | - 109 Other cranial puncture - 110 Intracranial pressure monitoring - 116 Intracranial oxygen monitoring - 121 Incision and drainage of cranial sinus - 123 Reopening of craniotomy site - 124 Other craniotomy - 125 Other craniectomy - 3881 Other surgical occlusion of vessels, intracranial vessels | 9 |
| Brain bleed word | - “IPH” - “aneurysms” - “embolize” | 9 |
| Brain word | - “*occipital” - “*cranio*” - “*cepha*” - “mening*” - “*frontal” - “*tempero*” - “*pariet*” - “brain” - “*arachnoid” - “mca” - “hemiparesis” - “hemiplegia” | 9 |
| Brain ischemia diagnosis code | - 3481* Anoxic brain damage - 434** Occlusion of cerebral arteries - 435** Transient cerebral ischemia - 4371* Other generalized ischemic cerebrovascular disease - 4376* Nonpyogenic thrombosis of intracranial venous sinus | 9 |
| Brain ischemia procedure code | - 62 Percutaneous angioplasty of intracranial vessel(s) - 65 Percutaneous insertion of intracranial vascular stent(s) - 116 Intracranial oxygen monitoring - 1754 Percutaneous atherectomy of intracranial vessel(s) - 3811 Endarterectomy, intracranial vessels | 9 |
| Brain trauma diagnosis code | - 3485* Cerebral edema - 3484* Compression of brain - 34939 Other dural tear - 800** Fracture of vault of skull - 801** Fracture of base of skull - 803** Other and unqualified skull fractures - 804** Multiple fractures involving skull or face with other bones - 850** Concussion - 851** Cerebral laceration and contusion - 852** Subarachnoid subdural and extradural hemorrhage following injury - 853** Other and unspecified intracranial hemorrhage following injury - 854** Intracranial injury of other and unspecified nature | 9 |
| Brain trauma procedure code | - 109 Other cranial puncture - 110 Intracranial pressure monitoring - 116 Intracranial oxygen monitoring - 123 Reopening of craniotomy site - 124 Other craniotomy - 125 Other craniectomy - 202 Other craniectomy | 9 |
| Leaky surgical wound word | Text has "surg*" and "wound" and ("drain*" or "leak*”) | 10 |
| Long stay | >9 days in hospital admission | 10 |
| Wound catheter word | - “catheter” - “placed” - “large” | 10 |
| Allergy or anaphylaxis word | - “allerg*” - “anaphyl*” | 11 |
| Drug AE code | 960** to 979** Poisoning By Drugs, Medicinals And Biological Substances | 11 |
| Surgery or medical AE code | 996** to 999** Complications Of Surgical And Medical Care, Not Elsewhere Classified | 11 |

Table S5. Summaries of the admissions with the top three topic match scores, for the most common topics for the time-based case. “Intubated” and “extubated” refer to starting and ending mechanical ventilation. AF: atrial fibrillation. ARF: acute renal failure. CABG: coronary artery bypass graft. CCU: critical care unit. CPR: cardiopulmonary resuscitation. DVT: deep vein thrombosis. HD: hospital day. IABP: intra-aortic balloon pump. O2: oxygen. tPA: tissue plasminogen activator. UTI: urinary tract infection.

| Topic #: Top 20 substantive terms | Summary of records with top 3 topic scores | Comment |  |
| --- | --- | --- | --- |
| Topic 18: for, hr, plan, vent, intubated, cont, today, skin, are, family, per, support, increased, off, goal, iv, placed, trach, foley, pain | Admitted on hospital day 1 (HD1) from other hospital, with end stage liver disease, now short of breath. Intubated. Pneumonia. Developed bacteremia. Coagulopathy and anemia due to liver. HD29 severe hypotension, extubated, comfort measures only, died. | Long complex stay |  |
|  | Admitted HD1 after CPR and intubation. No anticoagulants given; edema. Anemic initially. HD2 multiple chest fractures from CPR. HD1 to HD4, HD7 to HD15, HD16 to HD33 intubated; HD34 to HD35 O2 mask. HD2, HD35 hypotension. HD2 to HD36 AF. HD2 to HD7 pulmonary edema. HD9 surgery on spine; 4 units blood; postoperation hypotension. HD13, HD16, HD20, HD25, HD31 blood transfused. HD16 platelets dropped, stayed low despite removal of all heparin and heparin lines and despite daily platelet transfusions on HD16 to HD34. HD25 heparin-induced thrombocytopenia positive. HD18 edema increased. HD29 bone marrow biopsy. Died HD36. | Long complex stay |  |
|  | Admitted HD1 with ongoing anemia. Diagnosed leukemia. HD24 tooth pain, extracted, followed by intense pain and treated with antibiotics. Hepatitis B and C diagnosed. Blood transfusions. Chemotherapy. Bacteremia diagnosed and treated; other infections diagnosed over time; several antibiotics tried. HD49 bone marrow transplant. [Immunosuppressant] started, seemed to cause hypertension. HD76 to HD110 intubated. HD76 pulmonary edema. Progressive renal failure, treated with continuous dialysis HD88 to HD98. Died HD111. | Long complex stay |  |
|  |  | | |
| Topic 3: for, hr, pain, bp, are, you, iv, family, time, ccu, per, sats, note, heart, micu, received, skin, if, acute, plan | Admitted HD1 for chest pain. Inserted stent; then worse heart beat profile. HD2 went to CCU. Kidney worsened. Discharged HD8. | Heart attack, cardiac catheterization, heparin AE |  |
|  | Transfused monthly before admission. Admitted HD1 for declined mental status; diagnosed heart attack; started aspirin; not a cardiac catheterization candidate. HD1 started breathing difficulty; new tachycardia. HD2 pulmonary edema; hypotension observed and treated; AF; mask O2. HD3 to HD5 hypotension. HD3 to HD5 pulmonary edema. HD4 to HD? given blood. HD2 to HD4 given heparin. HD1 to HD4 fever. Discharged HD6. | Heart attack, (not) cardiac catheterization, heparin AE |  |
|  | Admitted HD1 for hypoxia and ARF; diagnosed renal cysts, new AF, UTI. HD1 to HD6 UTI. HD? ARF resolved. Discharged HD10. | Hypoxia and kidney failure |  |
|  |  | | |
| Topic 19: for, are, pain, you, comparison, acute, upper, evaluate, iv, trauma, hospital, if, note, time, large, level, pleural, wbc, read, throughout | Admitted HD1 for [event02]. Diagnosed fractures, dislocations, and muscle injury. Treated with [device02], [device03] and [device04]. Started antihypertensive drug. Discharged HD10 to home. | Bone trauma |  |
|  | [Event03] and went to other hospital. HD1 transferred tothis hospital; diagnosed with fractures; treated with [device02]. Diagnosed chronic kidney failure. Discharged HD4 to home. | Bone trauma, kidney failure |  |
|  | Admitted HD1 for abdominal pain from [event04]. Diagnosed spleen laceration. Discharged HD3. | Spleen trauma |  |
|  |  | | |
| Topic 7: for, are, pain, pleural, cabg, hr, plan, per, comparison, off, bp, pericardial, time, neo, iv, heart, md, mm, mr, catheter | Admitted HD1 for shortness of breath; to get cardiac catheterization. HD4 heart valve replaced; then hypotension treated with [phenylephrine] until next day. HD5 to HD8 heart rhythm abnormal. HD8 moved to step down. HD11 discharged. | Heart failure, cardiac catheterization, heparin AE |  |
|  | HD1 admitted for heart problem. Started heparin. HD4 replaced heart valve, CABG, placed IABP, started epinephrine, started levophed, gave blood. HD5 stopped IABP, epinephrine, levophed. HD6 new AF. HD7 went to floor. HD12 discharged. | Heart failure, cardiac catheterization, heparin AE |  |
|  | HD1 admitted for heart surgery; CABG; AF during operation; given [phenylephrine]; ongoing diabetes mellitus, type 2. HD2 went to floor. HD3 went to CCU to restart insulin drip. HD5 went to floor. HD9 discharged. | Heart failure, cardiac catheterization, heparin AE |  |
|  |  | | |
| Topic 1: for, are, family, subarachnoid, mm, comparison, pain, iv, occipital, sdh, large, evaluate, plan, cont, acute, craniotomy, per, hr, note, goal | HD1 admitted for headache and confusion; diagnosed brain bleed. Blood removed in operating room. HD2 moved to floor. HD4 same place in brain seen to still bleed; in operating room removed new blood and stopped bleeding. HD5 went to floor. HD10 discharged. | Brain bleed, brain surgery |  |
|  | Admitted HD1 for brain surgery. HD2 went to floor. Discharged HD3. | Brain surgery |  |
|  | HD1 transferred from other hospital that diagnosed brain bleed. HD2, HD3 surgery to remove blood from brain. HD6 went to floor. HD8 Magnetic resonance image, then seizures for an hour and moved to CCU. HD13, HD14 seizure-free. HD15. | Brain bleed, brain surgery |  |
|  |  | | |
| Topic 4: catheter, pleural, for, pain, jp, [pain reliever], placed, large, into, pigtail, hr, cont, french, increased, are, pseudoaneurysm, upper, skin, iv, comparison | HD1 admitted for large drainage from surgery; given fluids; drain replaced. HD4 peripherally inserted central catheter line placed for intravenous fluid. HD25 sclerotherapy to try to stop the leaking. HD34 surgery to stop leak. HD45 stent placed; drainage decreased. HD52 stent migrated and was removed; more stents placed. HD56 discharged to home to care for continuing drainage. | Extensive prolonged drainage after abdominal surgery |  |
|  | HD1 admitted due to [condition10] diagnosed at other hospital; pulmonary emboli and DVT; heparin started and stopped; AF. HD2 venous filter placed; bilateral pleural effusions; pulmonary embolism, DVT. HD3 catheter inserted in [condition10], turned out to be infected; started antibiotic. HD4 needed extra fluid; continuing AF; edema; stopping heparin. HD5 started O2 mask; AF; edema; pleural effusions bigger; pleural drain placed. HD6 catheter upsized; AF; edema improved. HD7 heparin; AF. HD10 catheter upsized. HD20 discharged to extended care; needs heparin lock flushes of catheter. | Extensive drainage of abdominal infection, already had pleural effusions, thrombi, and AF |  |
|  | HD1 transferred from other hospital for [condition11] and nearby fluid removal. HD2 inserted drain. HD4 new drain inserted. HD5 stent. HD8 another drain. HD13 fluids decreased; pleural effusions decreased. HD18 hypotensive; septic; drainage increased; antibiotics started. HD23 catheter repositioned. HD26 discharged. | Extensive drainage of abdominal organ, infection |  |
|  |  | | |
| Topic 17: for, are, mca, into, time, catheter, arteriogram, occlusion, mm, acute, french, ica, iv, placed, territory, large, cont, comparison, goal, family | HD1 transferred from other hospital for stroke; had received tPA. HD2 large brain bleeds. HD3 died. | Brain ischemia; brain bleed |  |
|  | HD1 transferred from other hospital where taken for signs of stroke; diagnosed brain arteries blocked. HD1 intubated; catheterization lab cleared thrombus; placed stent; antihypertensive after. HD2 extubated. HD9 discharged. | Brain ischemia |  |
|  | HD1 admitted for stroke symptoms; given tPA; an hour intubated and sedated, stented. HD2 extubated. HD3 went to floor. HD8 discharged. | Brain ischemia |  |
|  |  | | |
| Topic 12: [condition01], section, gynecology, [condition02], dystrophy, cesarean, [anti-thyroid], transabdominal, [event01], lmp, wk, [procedure01], [progesterone], prenatal, [condition03], [condition04], [antispasmodic], enteropathy, [condition05], [condition06] | HD1 admitted for abdominal pain; diagnosed with [condition01]; [device05] placed. HD2 [device05] removed; [condition01] resolved. HD3 [condition01] returned; [device05] placed again. HD5 suddenly needed O2. HD6 to HD8 antibiotics for UTI. HD11 discharged. | [Condition01] |  |
|  | HD1 admitted for [condition01]; open surgery to resolve it; also [procedure02]. HD10 discharged. | [Condition01] |  |
|  | HD1 admitted for distressing symptoms; diagnosed [condition01]. [Procedure03] temporarily resolved the condition. HD5 [Procedure04] resolved [condition10]. HD13 discharged. | [Condition01] |  |

Table S6. Summaries of documents (top scoring for 20-11 and all for the other topics) with topic matching scores for the less common topics in the time-based case. “Rare” means there were no instances in period 1 “Unusual” means there were a few or some instances in period 1.. CCU: critical care unit. PICC: peripherally inserted central catheter

| Topic #: top 20 substantive terms | [Topic match score] Brief summary of text | Topic fit | AE type | Text offers more AE data than codes? |  |
| --- | --- | --- | --- | --- | --- |
|  |  | | | | |
| Topic 11: pentobarb, pentobarbital, cmv, encasement, prison, [condition07], satellite, hematologic, rent, [condition08], [condition09a], [condition09b], [antibiotic], federal, bleach, [device01], allergic, [rare-word01], cluster, [rare-word02] | [0.11] Admitted HD1 due to [event05]. Given anaphylaxis meds. Discharged HD2. | "Allergic" is more common in the post period. | Non-medical anaphylaxis. | No |  |
|  | [0.08] Admitted HD1 with recent [event06]. Diagnosed [condition9b]. Started antibiotic and developed [condition12], thought to be [condition13] and treated with drugs for [condition13]. Also given [antiviral]. Diagnosed [condition14] so stopped prior antibiotics. In CCU started therapy for [condition14]. HD5 given PICC line for that therapy. Discharged HD6. | “[Condition9a]” and “[condition9b]” are both rare in the text. | Medical therapy AE. | Yes |  |
|  | [0.08] Admitted HD1 due to [event07]. HD2 procedure resolved [event07]; discharged. [No discharge summary] | “[Event07]” is rare in the text. |  |  |  |
|  | [0.08] Admitted HD1 for surgery for [condition07]; had surgery and chest tubes. HD2 transferred to floor. Tubes gradually removed. Discharged HD13. | “[Condition07]” is unusual in text. |  |  |  |
|  | [0.08] Admitted HD1 due to [condition07]; had surgery, drainage tubes, and epidural. HD2 postsurgical [device06] malfunctioned so replaced; tachycardia; on heparin prophylaxis. HD3 replacement [device06] failing the same way as the first. HD5 epidural removed. HD7 one of the drains removed. Discharged HD8. | “[Condition07]” is unusual in text. | Medical therapy AEs. | Yes, and more information in daily notes than discharge summary. |  |
|  |  | | | | |
| Topic 5: [rare words, misspelled words] | [0.05] Admitted HD1 from other hospital for [condition15] and [condition16]. The latter gradually decreased during the stay. HD7 new [condition17]. Discharged HD18. New [condition17] thought to be side effect of combination of therapies; new [condition17] gradually improved. | “[Condition15]” is unusual in the text. | Medical therapy AE. | Codes indicate the outcome, but not the speculated causes. |  |
|  |  | | | | |
| Topic 15: [rare words, misspelled words] | [0.13] Admitted HD1 due to reaction to [drug01]. Treated. Discharged HD2. | “[Drug01]” is an unusual word in text. | Medical therapy AE. | Codes say reaction to named drug. |  |
|  | [0.13] Admitted HD1 for distressing symptoms. Diagnosed many conditions. Family refused aggressive treatment. HD13 went to floor. Discharged HD16. | “[Rare-word03]” is rare in the text. |  |  |  |
|  |  | | | | |
| Topic 16: [rare words, misspelled words] | [0.11] Admitted HD1 due to [condition18a and b] known diabetes mellitus, type 1 and hypothyroid; [procedure01] done to treat. HD2 orthostatic hypotension. HD3 blood pressure fine; discharged. | “[Condition18a]” and “[condition18b]” are both rare in the text. |  |  |  |
|  | [0.05] Admitted HD1 from outpatient clinic where had received [drug02], then needed rescue. AE attributed to [drug02]. Continued to improve. Discharged HD3. | “[Drug02]” is rare in the text. | Medical therapy AE. | There is a code for this AE, but not clear which of the other codes is the actual AE. |  |
|  |  | | | | |
| Topic 10: [rare words, misspelled words] | [0.06] Admitted HD1. [Only 2 notes, both nursing]. Bleeding in brain. Discharged HD2. | “[Rare-word04]” is rare in text and was quoting the patient. |  |  |  |
|  | [0.04] Admitted HD1 incoherent, prior [condition19a and b], other diseases. Has pneumonia. HD2 coherent. Discharged HD7. | “[Condition19a]” and “[condition19b]” are rare in the text. |  |  |  |
|  |  | | | | |
| Topic 0: [rare word] | [0.04] Admitted HD1 from other hospital for [event08] following [procedure05]. Started antibiotics. HD2 improved; moved to floor. Discharged HD6. | "[Procedure05]" is unusual in the text. | Medical therapy AE. | Codes specify drug AE, and hint, but don't specify the surgical procedure. |  |
|  |  | | | | |
| Topic 2: [rare words, foreign language words, misspelled words] | [0.12] [Patient instructions are in foreign language. Rest of record is in English.] | Foreign language words are unusual or rare. | Medical therapy AE. | Outcomes are in the billing codes but codes don't indicate that one might be a medical therapy AE. |  |
|  | [0.08] [Patient instructions are in foreign language. Rest of record is in English.] | Foreign language words are unusual or rare. |  |  |  |
|  | [0.03] [No foreign language.] Admitted HD1 for [event09] that resulted from loss of consciousness; several fractures and cuts. Cuts sewn. [Device02], [device07] and [device08]; given. Discharged HD4. | "[Rare-word05]" and "[rare-word06]" are rare in the text. |  |  |  |
|  |  | | | | |
| Topic 14: [rare words, misspelled words] | [0.03] Admitted HD1 for [procedure06] for [condition20]. HD3 went to floor. HD10 new fever; diagnosed bacterial infection in surgical drainage; given antibiotics. Fistula noted. Discharged HD43. | "[Procedure06]" and "[condition20]" are rare words in text. | Medical therapy AEs. | The order and consequences of events are not noted by the codes. |  |
|  |  | | | | |
|  | Topic 9: [rare words, misspelled words] | | | | |
| Topic 9: [rare words, misspelled words] | Admitted HD1 to treat [condition14]; PICC inserted; treatment was uneventful. HD2 began next phase of therapy; went to floor. HD5 started warmth and redness near PICC line insertions site. HD6 worse; no thrombus found; treated with warm compresses. Discharged HD8 to extended care for continued therapy. Heparin for prophylaxis. | "[Condition14]" unusual in text. | Medical therapy AE. | In discharge summary but not in codes. |  |
|  | Admitted HD1 with recent[event06]. Started antibiotic and developed [condition12], thought to be [condition13], and treated [condition13]. Also given antiviral. Diagnosed [condition14] so stopped prior antibiotics. In CCU started [condition14] therapy. HD5 given PICC line for continued treatment. Discharged HD6. | "[Condition14]" unusual in text. | Medical therapy AE. | In discharge summary but not in codes. |  |
|  |  | | | | |
|  |  | | | | |
| Topic 13: [rare words, misspelled words] | HD1 had [event10a]; no fracture. Precautionary CCU, showed no issue. HD1 discharged. [No discharge summary.] | "[Event10a]" and "[event10b]" are rare words in the text. |  |  |  |
|  | Admitted HD1 for lightheaded, stomach pain. Anemia. Diagnosed upper gastrointestinal tract ulcer and treated; may have been worsened because of prescribed high [blood thinner] doses. Transfused. Discharged HD6 to home. | "[Rare-word07]" is rare in the text. | Medical therapy AE. | No |  |
|  | Admitted HD1 due to [event02] and lost consciousness; multiple fractures, brain bleed. Discharged HD5. | "[Rare-word08]" is rare in the text. |  |  |  |
